# Oncolytic Viruses: Immunotherapy Drugs for Gastrointestinal Malignant Tumors

**DOI:** 10.3389/fcimb.2022.921534

**Published:** 2022-06-03

**Authors:** Qingbo Li, Patrick Kwabena Oduro, Rui Guo, Ruiqiao Li, Ling Leng, Xianbin Kong, Qilong Wang, Long Yang

**Affiliations:** ^1^College of Traditional Chinese Medicine, Tianjin University of Traditional Chinese Medicine, Tianjin, China; ^2^Research Institute of Traditional Chinese Medicine, Tianjin University of Traditional Chinese Medicine & State Key Laboratory of Component-Based Chinese Medicine, Ministry of Education, Tianjin, China; ^3^Research Center for Infectious Diseases, Tianjin University of Traditional Chinese Medicine, Tianjin, China; ^4^School of Integrative Medicine, Tianjin University of Traditional Chinese Medicine, Tianjin, China

**Keywords:** oncolytic virus, digestive system, immunotherapy, cancer, tumor

## Abstract

Oncolytic virus therapy has advanced rapidly in recent years. Natural or transgenic viruses can target tumor cells and inhibit tumor growth and metastasis in various ways without interfering with normal cell and tissue function. Oncolytic viruses have a high level of specificity and are relatively safe. Malignant tumors in the digestive system continue to have a high incidence and mortality rate. Although existing treatment methods have achieved some curative effects, they still require further improvement due to side effects and a lack of specificity. Many studies have shown that oncolytic viruses can kill various tumor cells, including malignant tumors in the digestive system. This review discusses how oncolytic virus therapy improves malignant tumors in the digestive system from the point-of-view of basic and clinical studies. Also, the oncolytic virus anti-tumor mechanisms underpinning the therapeutic potential of oncolytic viruses are expounded. In all, we argue that oncolytic viruses might eventually provide therapeutic solutions to malignant tumors in the digestive system.

## 1 Introduction

Gastrointestinal malignancies’ mortality rate remains high, with gastric and colorectal tumors among the top five cancers with high incidence rates worldwide (Sung et al., 2021). For decades, a growing list of different treatment options to control gastrointestinal cancers has been emerging. The main treatment choices are chemotherapy, radiotherapy, radiofrequency ablation, and surgical resection. However, these treatment options have a number of drawbacks, including severe side effects, inability to complete resection, and resistance to long-term use of drugs. This shows that effective treatments with profound tumor-carnage properties and low adverse and side effects are needed.

Oncolytic viruses are usually natural or modified viruses that target and kill tumor cells. These viruses regulate key intracellular processes and anti-viral responses such as apoptosis, inflammation, angiogenesis, and the cell cycle (Chen et al., 2021). Also, oncolytic viruses can enhance the host’s anti-tumor immunity through multiple mechanisms (Ramelyte et al., 2021). The ability of oncolytic viruses to regulate the above-stated intracellular processes and anti-viral events, as well as their capacity to be transformed to express tumor-destructive factors, makes them an effective anti-tumor agent of great clinical potential. In addition, compared to other anti-tumor agents, oncolytic viruses generally have outstanding characteristics, such as being non-pathogenic, having a relatively good safety profile, and the ability to be engineered to destroy tumor cells but not healthy cells, as well as the ability to deliver therapeutic payloads and produce immune-boosting molecules specific to the tumor cells they infect (Lawler et al., 2017). Because of these unique features, oncolytic virotherapy–generally defined as a treatment option that uses oncolytic viruses to kill cancer cells–has emerged as a promising therapeutic approach to treat cancers, including malignant tumors of the digestive system (Cook and Chauhan, 2020).

On the therapeutic front, critical breakthrough lenses that have emerged are the need to optimize oncolytic virotherapy to modulate the tumor immune microenvironment and combine oncolytic virotherapy with other immunotherapies or anticancer treatment options to derive maximal clinical benefit. Therefore, this mini-review provides a comprehensive overview of the development and application of oncolytic virus immunotherapy alone and/or in combination with other therapies to treat malignant tumors of the digestive system. We also discuss preclinical and clinical studies supporting oncolytic viruses’ role in gastrointestinal malignant tumor therapy and detail the unique therapeutic mechanisms modulated by oncolytic viruses against cancers.

## 2 Anti-Tumor Mechanism of Oncolytic Virus

### 2.1 Direct Oncolysis

**Figure 1:**
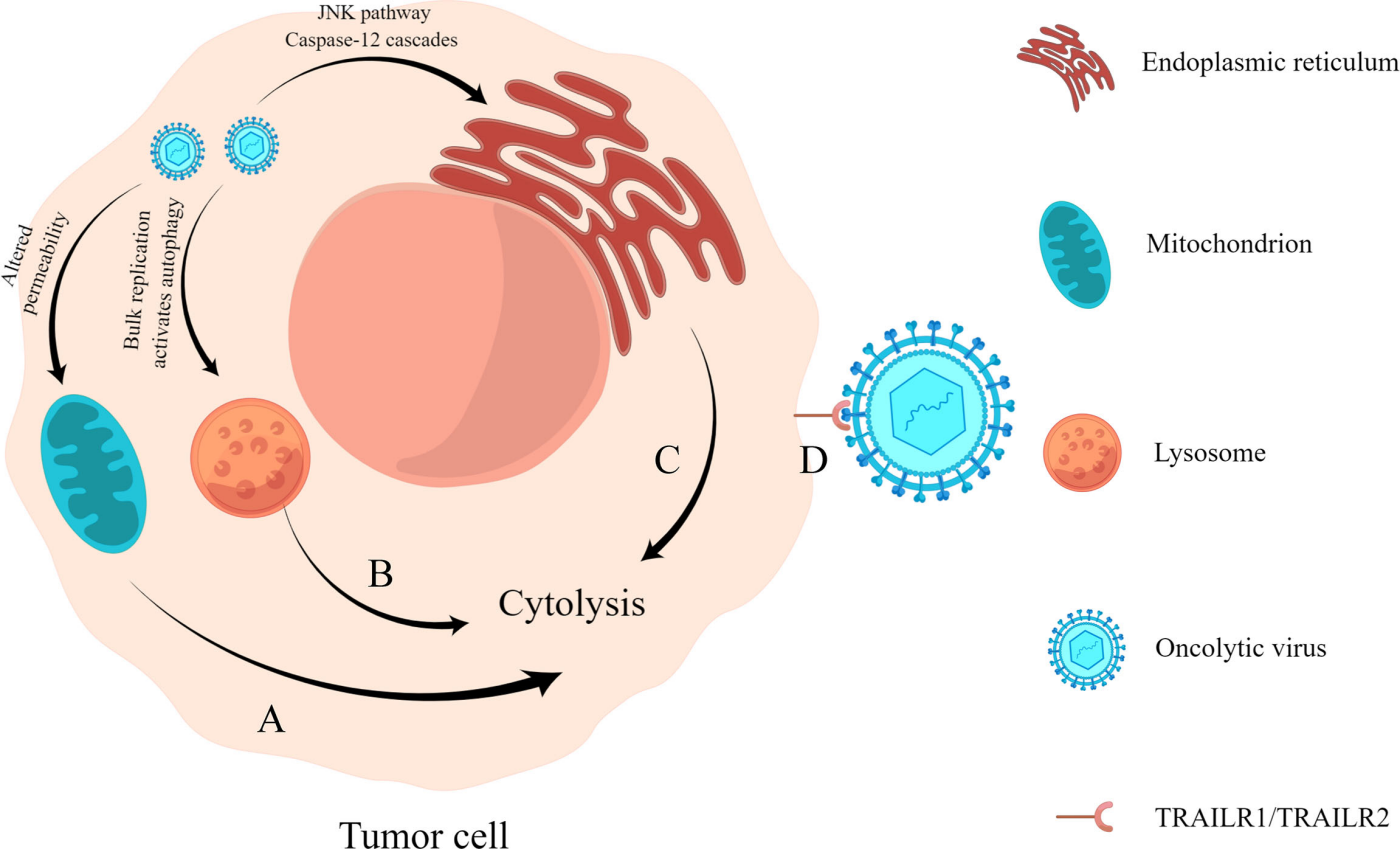
Direct oncolysis. **(A)** Oncolytic virus acts on the mitochondria of tumor cells, resulting in the loss of mitochondrial permeability, resulting in abnormal cell metabolism and death. **(B)** Oncolytic viruses replicate abundantly and cause lysosomes to become damaged, triggering cell lysis (Yang et al., 2021). **(C)** Oncolytic virus infection causes endoplasmic reticulum stress response and cell lysis by activating the JNK pathway and caspase-12 cascades. **(D)** Virus surface capsid protein binds to TRAILR1 or TRAILR2 and causes apoptosis through the molecular mechanism of trail-mediated apoptosis.

When an oncolytic virus infects and replicates in tumor cells, it affects the synthesis of nucleic acid and protein in cells and damages organelles such as lysosomes, endoplasmic reticulum, and mitochondria, leading to alterations in cell function and, finally, killing tumor cells (**Figure 1**). For example, the recombinant Newcastle disease virus R2B-GFP virus causes the loss of mitochondrial membrane permeability in 4T1 and B16-F10 cells, resulting in cell death (Ramamurthy et al., 2021). The M1 virus kills cancer cells by inducing endoplasmic reticulum stress-mediated apoptosis (Lin et al., 2014). In addition, the capsid protein of an oncolytic virus can also play a direct role in oncolysis (Yang et al., 2021). Engineered A4 virus carrying the TRAIL gene expresses TRAIL protein on the viral surface by linking to the Leu zipper of capsid protein IX (Wang et al., 2016), which can bind to its receptor TRAILR1 (also known as DR4) or TRAILR2 (also known as DR5) to specifically induce apoptosis in cancer cells (Johnstone et al., 2008).

### 2.2 Inhibition of Intra-Tumor Angiogenesis

Angiogenesis plays an important role in tumor growth and development (Lugano et al., 2020). Many studies have shown that an oncolytic virus can effectively inhibit tumor angiogenesis and limit the supply of oxygen and nutrients to tumor cells. As a result, tumor cells are eliminated (Gholami et al., 2016), while at the same time, tumor cell proliferation is prevented (Qian and Pezzella, 2018).

**Figure 2:**
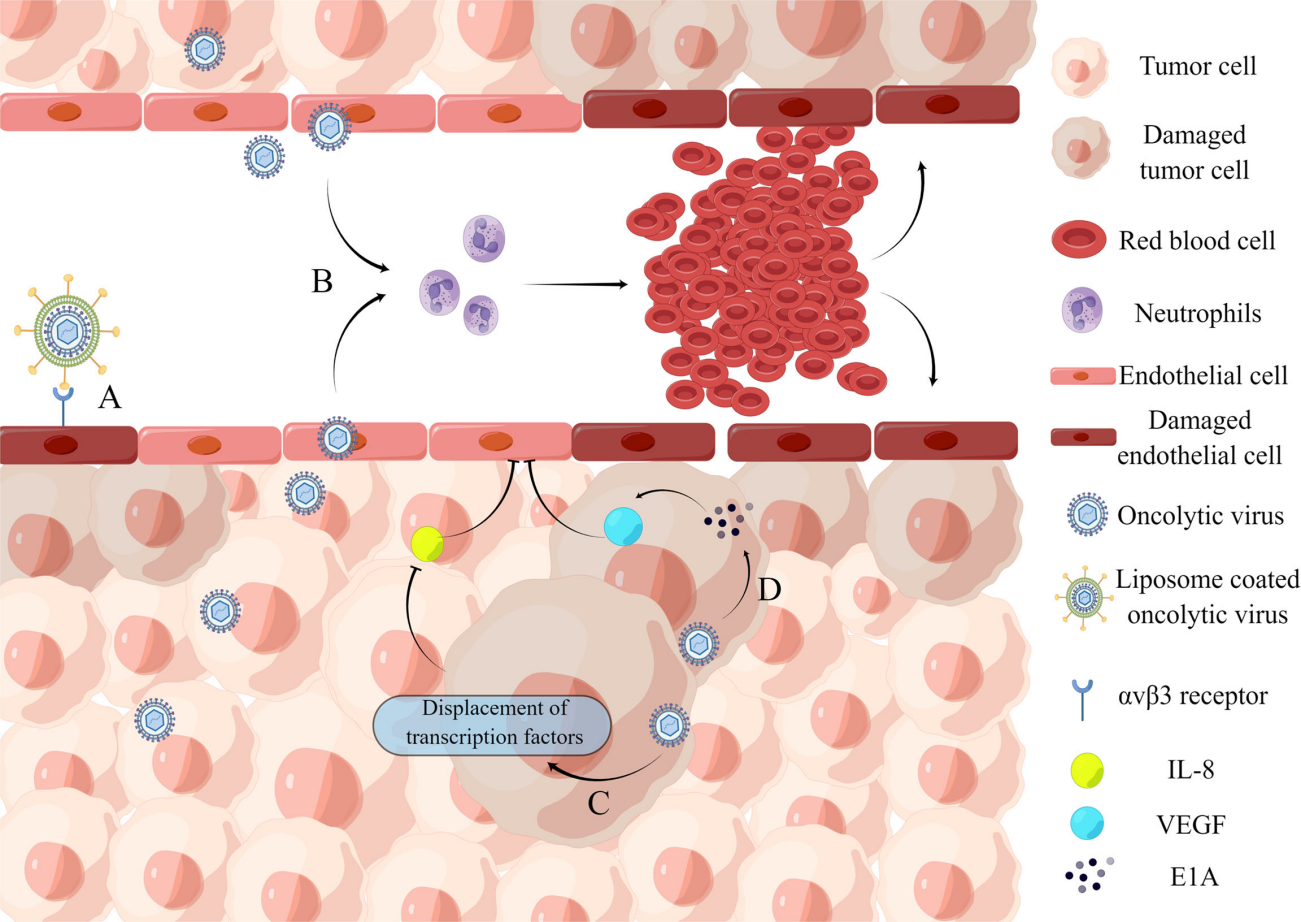
Inhibition of intra-tumor angiogenesis. **(A)** Binding of iRGD liposome-encapsulated oncolytic virus to αvβ3 receptor directly induces lysis of HUVECs. **(B)** Oncolytic virus infection causes the recruitment of a large number of neutrophils and the formation of microthrombosis, resulting in the loss of blood perfusion and the increase of tumor cell apoptosis caused by ischemia. **(C)** Oncolytic virus dl922-947 treatment reduces IL-8 production in ATC cell lines by displacing the transcription factor NF-κB p65 from the IL8 promoter, thereby inhibiting tumor angiogenesis. **(D)** Adenovirus can express E1A protein, which can downregulate VEGF by interacting with angiogenic proteins, thereby affecting neointima in the tumor microenvironment and ultimately achieving tumor lysis.

Tumor angiogenesis can be influenced by oncogene-mediated protein expression and cellular stress factors such as hypoxia, low pH, nutrient deficiency, or reactive oxygen species induction (Al-Ostoot et al., 2021). An oncolytic virus can play an anti-angiogenesis role in many ways (Angarita et al., 2013) (**Figure 2**): (1) Direct infection of tumor vascular cells leads to the lysis of vascular endothelial cells; (2) Induce virus-mediated immune response, resulting in cell aggregation and reduced tumor blood flow; (3) Express viral proteins with anti-angiogenesis properties or inhibit the synthesis of angiogenesis promoting factors.

A recent study demonstrates the ability of an oncolytic virus to lyse vascular endothelial cells. The iNDV3α-LP binds to tumor neovascularization *in-vivo* and promotes endothelial cell lysis (Wang et al., 2022). And the effect on blood flow is mainly through the recruitment of neutrophils. VSV infection of tumors causes massive neutrophil infiltration, resulting in loss of perfusion due to ischemia, which leads to increased apoptosis of tumor cells (Breitbach et al., 2007). When cancer progresses, the concentration of anti-angiogenic factors decreases. Endothelial cell growth and migration are stimulated by vascular endothelial growth factor (VEGF), epidermal growth factor (EGF), fibroblast growth factor (FGF), and interleukin-8 (IL-8) (Jośko et al., 2000). For example, an oncolytic adenovirus impairs IL-8-induced angiogenesis in pancreatic cancer (Passaro et al., 2016). Furthermore, several oncolytic herpesviruses are engineered to produce angiostatin, which has anti-angiogenic properties in a range of tumor models (Ye et al., 2006; Nair et al., 2021). However, some oncolytic viruses, such as herpesvirus C-REV, enhance tumor angiogenesis rather than inhibit it (Aghi et al., 2007; Kurozumi et al., 2008; Sahin et al., 2012). Therefore, caution should be exercised concerning their use in oncolytic virotherapy. However, combining pro-angiogenic oncolytic viruses with anti-angiogenic strategies can improve their efficacy.

### 2.3 Regulation of Anti-Tumor Immunity

**Figure 3:**
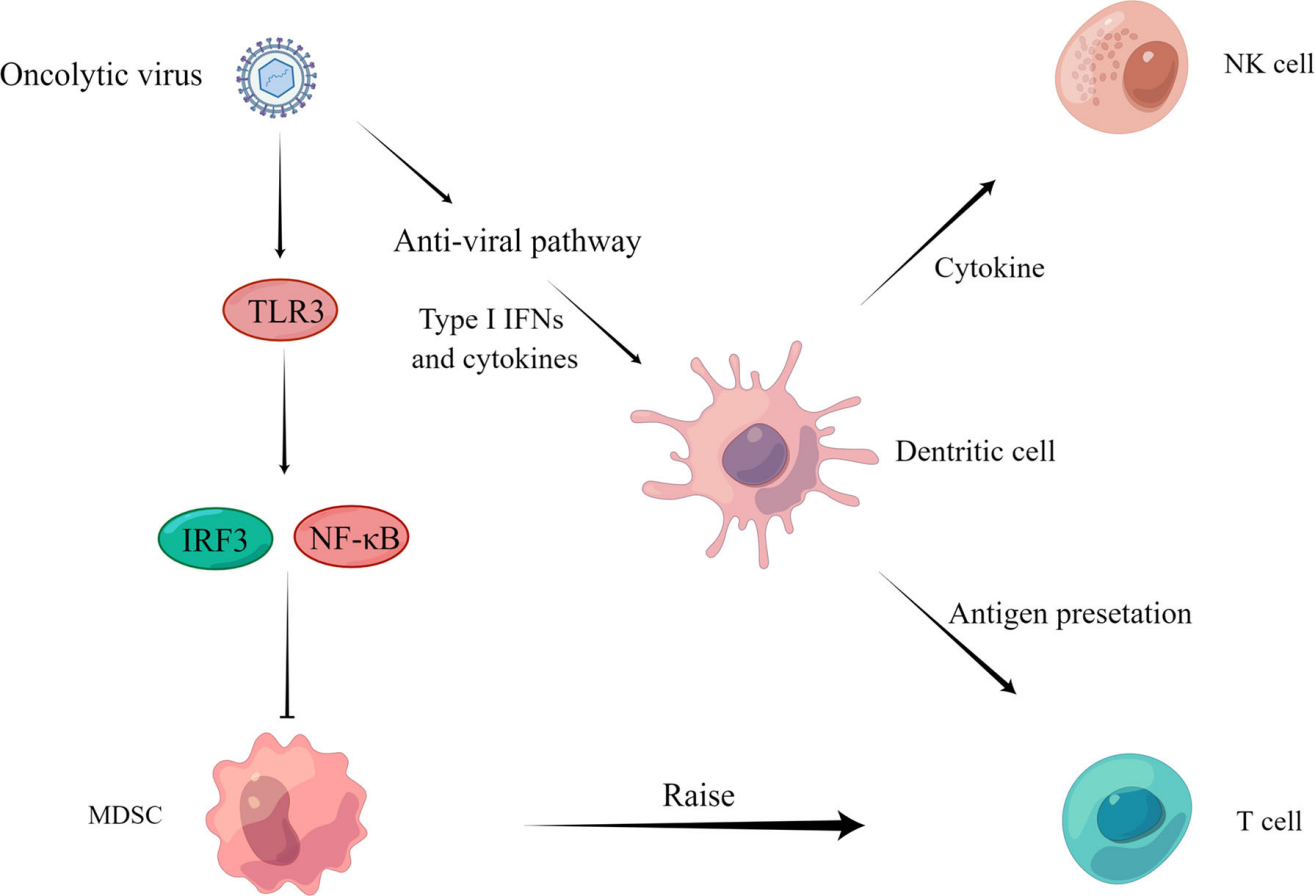
Regulation of anti-tumor immunity. On the one hand, the virus activates IRF3 or NF-κB through the TLR3 pathway and inhibits the proliferation of MDSCs (Katayama et al., 2018) to recruit T cells to play an immune role. On the other hand, the virus activates the anti-viral pathway, induces the production of type I IFN and other cytokines, acts on DCs, NK cells, and T cells through cytokine and antigen presentation, and finally activates the body’s immune response and kills tumor cells.

The expression of immunosuppressive cytokines in tumor cells and tumor immune microenvironment is linked to the inactivation of effector immune cells and even recruitment of immunosuppressive cells, resulting in the body’s inability to clear tumor cells in this immunosuppressive state (Borsig, 2018; Axelrod et al., 2019). Therefore, changing the suppressed state of the tumor immune microenvironment has an essential role in clearing tumor cells. Oncolytic viruses can alter the cytokine milieu and enhance immune cell maturation and activation, restoring and increasing the body’s function in tumor cell clearance (Raja et al., 2018; Galon and Bruni, 2019) **(** **Figure 3****)**.

An oncolytic virus can play an anti-tumor role by reversing the immune silencing state of the tumor immune microenvironment. For example, OHSV2 can effectively reduce the levels of bone marrow-derived suppressor cells (MDSCs) and regulatory T cells (Tregs) in the spleen, thus achieving reversal of the immunosuppressed state (Zhang et al., 2020). In addition, treatment of homozygous mice bearing 4T1 TNBC tumors with G47Δ-mIL12 was able to reduce MDSCs, leading to CD8 T cell-dependent inhibition of 4T1 tumor growth, inhibition of tumor angiogenesis, and prevention of lung metastasis, while increasing intra-tumor CD8 T cell infiltration, with local and systemic anti-cancer effects (Ghouse et al., 2020).

Tumor killing is aided by natural killer cells (NK), CD8 T cells, and dendritic cells (DC). Oncolytic viruses have been proven in numerous investigations to activate or recruit these immune cells to have an anti-tumor effect. The levels of NK cells, CD8 T cells, and DC increased dramatically after OHSV2 treatment (Ghouse et al., 2020). This treatment response was equated to being the mechanism by which OHSV2 exerts its oncolytic activity. In addition, oncolytic herpes simplex virus (T-VEC) can recruit tumor antigen-specific CD8 T cells and induce anti-inflammatory gene characteristics in injected and non-injected tumors (Ye et al., 2018). Furthermore, Newcastle disease virus (NDV) infection stimulates type I interferon (IFN) production *via* other tumor-infiltrating immune cells (García-Romero et al., 2020), as well as type I IFN signaling for antigen presentation, DC maturation, memory cytotoxic T lymphocyte activation and survival, NK cell activation, and neutrophil recruitment (Zitvogel et al., 2015; Parker et al., 2016). Engineered lysing virus and Semliki Forest Virus (SFV) infections that express a programmed death ligand-1 (PD-L1) inhibitor, as well as a granulocyte-macrophage colony-stimulating factor (GM-CSF), have been shown to activate antigen-specific T cell (Ma et al., 2020; Wang et al., 2020).

Oncolytic virus therapy can establish long-term anti-tumor immunity in immune regulation and prevent tumor recurrence. For example, the M1 virus can destroy the immune tolerance of the tumor immune microenvironment, change the immunosuppressive state, trigger an effective CD8 T cell-dependent treatment effect, and establish long-term anti-tumor immune memory in a tumor model with poor immunogenicity (Liu et al., 2020). A study of the oncolytic vaccinia virus also illustrates this mechanism. The use of tumor-selective oncolytic vaccinia viruses encoding interleukin-7 (IL-7) and interleukin-12 (IL-12) can make mice with complete tumor regression resist the re-attack by the same tumor cells (Nakao et al., 2020).

## 3 Oncolytic Virus Immunotherapy in Gastrointestinal Malignant Tumors

### 3.1 Oncolytic Virus (Including Transgenic) Monotherapy

In this new era of innovative medicine, oncolytic viruses have become a promising primary treatment essential to fighting cancer. Various natural viruses have been found to have an effective oncolytic effect throughout many years of research. Natural viruses, on the other hand, have low specificity and pathogenicity, so researchers have been continuously optimizing them through transgenic technology to enhance their tumor specificity and selectivity. This section aims to focus on the latest advances in clinical applications of various oncolytic virus immunotherapies in the treatment of malignant tumors of the digestive system, especially when coupled with other cancer treatment strategies **(** **Table 1****)**.

**Table 1:** Oncolytic virus (including transgenic) monotherapy.

Type of cancer	Virus	Virus name	Route of virus administration	Effect	Reference
Colorectal cancer	CV	PD	Intratumoral injection.	Strongly inhibit tumor growth	(Hazini et al., 2018)
		PD-H	Intratumoral injection	Strongly inhibit tumor growth	(Hazini et al., 2021a)
		CVB3-375TS (3+)	Intratumoral injection	Reduced toxicity to pancreas	(Pryshliak et al., 2020)
		H3N-375TS	Intratumoral injection	Significantly slow down tumor growth	(Hazini et al., 2021b)
		H3N-375/1TS	Intratumoral injection	Significantly slow down tumor growth	(Hazini et al., 2021b)
	HSV	oHSV2	Intratumoral injection	Significantly slow down tumor growth	(Ying et al., 2017)
		oHSV2	Intratumoral injection	Effectively kill primary tumors and attack distal and metastatic tumors	(Zhang W. et al., 2021)
	VSV	ΔM51 mutant	Cell experiment	Significant cytotoxicity	(Gray et al., 2019)
	VV	VVLΔTKΔN1L-mIL-21	Intratumoral injection	Reduced toxicity to pancreas	(Wang et al., 2021)
Liver cancer	ARV	ARV-PB1	Cell experiment	Replicate normal and induce intense cytopathy	(Kozak et al., 2017)
	Influenza A virus	M1	i.v.	Reduced toxicity to pancreas	(Ying et al., 2017)
	AdV	Ad-VT	Intratumoral injection	Reduced toxicity to pancreas	(Tian et al., 2020)
	CV	NOV	Intratumoral injection	Reduced toxicity to pancreas	(Jeong and Yoo, 2020)
		CVV	i.p.	Effectively reduce cancer cell metastasis	(Yoo et al., 2017)
	Chimeric virus	rVSV-NDV	I.v.	Reduced toxicity to pancreas	(Abdullahi et al., 2018)
Pancreatic cancer	AdV	Ad5/3-E2F-d24-vIL2	Intratumoral injection	Significantly prolonged survival	(Quixabeira et al., 2021)
		LOAd703	i.p.	Significantly slow down tumor growth	(Eriksson et al., 2017)
		OBP-702	Intratumoral injection	Significantly inhibit the growth and invasion of cancer cells	(Koujima et al., 2020)
	OV	CF33-hNIS-antiPDL1	i.v.	Reduced toxicity to pancreas	(Woo et al., 2020)
	VSV	VSV-p53OV	Cell experiment	Normal replication and stable inheritance	(Steinhauer et al., 1992)
Gastric cancer	HSV	T-SOCS3	Cell experiment	Significant cytotoxicity	(Matsumura et al., 2021)
		G47Δ	Intratumoral injection /i.v.	Reduced toxicity to pancreas	(Sugawara et al., 2020)
	AdV	Ad-Surp-mK5/Ad-Surp-MnSOD 296	unknown	Reduced toxicity to pancreas	(Liu et al., 2022)
Oesophageal cancer	HSV	G47Δ	Intratumoral injection	Reduced toxicity to pancreas	(Yajima et al., 2021)

#### 3.1.1 Colorectal Cancer (CRC)

Coxsackievirus is a single-stranded RNA virus that belongs to the small ribonucleic acid family of human enteroviruses (Simmonds et al., 2020), among which coxsackievirus B3 (CVB3) has much great potential in the fight against colorectal cancer. However, wild-type (WT) CVB3 treatment leads to cardiac and pancreatic damage (Deng et al., 2019; Jia et al., 2019; Hazini et al., 2021b). As a result, strategies to improve its safety while also improving its therapeutic efficacy are required. First, one way is to select from numerous strains preferentially. Also, PD uses N- and 6-O-sulfonated acetylated heparin sulfate (HS) to enter host cells. It infects colorectal cancer cell lines with the highest efficiency *in-vitro* when compared to Nancy and 31-1-93 (Zautner et al., 2003). In addition to the good safety profile, *in-vivo*, PD application in a mouse colorectal cancer transplant tumor model showed significant tumor growth inhibition (Hazini et al., 2018). Second, another effective strategy is targeting the damage produced by CVB3 in the pancreas with transgenic modification of coxsackieviruses. For instance, researchers inserted miR-375TS into CVB3’s 3′UTR, a multi-protein coding sequence of CVB3, that reduced viral infection of pancreatic cells while maintaining CVB3’s tumor lytic function in an *in-vitro* experiment (Pryshliak et al., 2020). Of course, viral toxicity to the heart is non-negligible. In this case, combining the basal pancreas-specific expression of miR-375 with the heart-specific expression of miR-TS would enhance CVB3 virus effectiveness in preventing virus replication in the pancreas and heart while retaining the anti-tumor effect. This strategy further improves the specificity of CVB3 in animal experiments (Hazini et al., 2021b). It is worth noting that coxsackieviruses’ safety is tightly linked to the virus’s route of administration, and a recent study indicated that intratumoral injection of recombinant CVB3 variant PD-H caused no side effects. In immunocompetent mice, however, intraperitoneal administration resulted in weak pancreatitis and myocarditis (Hazini et al., 2021a).

In addition to CVB3, the herpes simplex virus (HSV) has shown tumor lysis potential. In 2015, the U.S. Food and Drug Administration (FDA) approved genetically modified T-VEC to treat advanced melanoma patients (Guo et al., 2019). In recent years, the herpes simplex virus has demonstrated therapeutic potential against various tumors (Hamada and Yura, 2020; Mondal et al., 2020; Bernstock et al., 2021). For example, HSV-2 expressing GM-CSF showed potent anti-tumor effects in multiple CRC cell lines and mouse CRC models, as well as the ability to modulate the immune response to enhance the therapeutic effect further. According to a recent animal study, HSV-2 expressing GM-CSF-induced immune response was effective against metastatic tumors, resulting in lasting anti-tumor effects and efficient prevention of tumor recurrence (Zhang W. et al., 2021). Furthermore, vesicular stomatitis virus (VSV) and its ΔM51 mutant exhibited a destructive effect on SW480 colorectal cancer cells in an *in-vitro* cellular study (Gray et al., 2019). VVLΔTKΔN1L-mIL-21, a novel lysing virus-containing IL-21, effectively induced adaptive T-cell responses that eliminated primary tumors and prevented tumor recurrence in an animal study (Wang et al., 2021). These studies, taken together, provide new viral options for the treatment of colorectal cancer. Aside from the anti-tumor effects, genetic modification of the virus can help us determine the appropriate dose for treatment. In complete and EEV forms, the recombinant virus CF33Fluc, which was obtained by substituting the thymidine kinase gene with firefly luciferase (Fluc), is more effective than the parental virus in another animal study. Real-time non-invasive imaging of viral replication can be used to assess viral replication in real-time (Warner et al., 2019).

#### 3.1.2 Liver Cancer

Hepatitis C virus (HCV) infection is common in people with liver cancer, and IFN is frequently used to treat it. As a result, treating liver cancer patients with an oncolytic virus will present two challenges: the first is determining whether the oncolytic virus can play a role in HCV-infected cells, and the second is determining whether interferon can inhibit the replication of the oncolytic virus. Researchers have recently looked into the two issues mentioned above. Studies have shown that avian reovirus ARV-PB1 can kill various liver cancer cell lines *in-vitro* (Kozak et al., 2017). In liver cancer cell lines infected with HCV, ARV-PB1 can still replicate normally and play a typical role in tumor lysis. Another study discovered that IFNα could activate IFN stimulating genes (ISGs), inhibit M1 virus replication, and prevent cell apoptosis. Thus, IFN treatment antagonizes the M1 virus’s oncolytic action. Other anti-hepatitis drugs, such as direct anti-viral small molecule drugs (DAA) and ribavirin (RBV) for chronic hepatitis C, do not inhibit M1’s oncolytic activity. As a result, the patient’s conditions must be evaluated before beginning IFNα and M1 virus combined therapy in patients with hepatocellular carcinoma complicated by hepatitis B virus or hepatitis B virus infection. When the expression of ISGs is abundant in the tumors of patients with liver cancer, coadministration of IFNα with M1 virus is not recommended. The standard chemical-based anti-hepatitis regimens should be selected in this case (Ying et al., 2017).

Various recombinant viruses have better tumor specificity, less toxicity to normal cells, and can inhibit tumors from metastasizing. In a tumor-bearing nude mouse model assay, the dual cancer-specific anti-tumor recombinant adenovirus Ad-apoptin-hTERTp-E1a (Ad-VT) showed tumor-specific replication and specific tumor-killing. In addition, in hepatocellular carcinoma QGY-7703 cells, it effectively inhibited tumor growth and promoted apoptosis (Tian et al., 2020). The NOV, which was obtained by inserting TRAIL and Ang1 into the engineered VV, also had anti-tumor effects *via* the apoptotic pathway in CRC homologous mouse models (Jeong and Yoo, 2020). The treatment of mice with *in-situ* hepatocellular carcinoma with the novel hybrid virus rVSV-NDV significantly prolonged the mice’s survival time. In addition, it had a reduced cytotoxic effect on healthy hepatocytes and neurons while retaining its benefits (Abdullahi et al., 2018). Furthermore, more than half of hepatocellular carcinoma patients develop metastases (Yoo et al., 2017), and oncolytic viruses have shown promise in the treatment of metastatic liver cancer. For instance, in an animal model of metastatic liver cancer established with a high CD44-expressing Sk-Hep-1 cell line, CVV, an engineered poxvirus produced by repeated selective replication in cancer tissue and deletion of the viral thymidine kinase gene, attenuates cell migration. Therefore, they reverse the metastasis of highly metastatic Sk-Hep-1 cells by inducing low CD44 to reduce the expression of EMT markers. This study provides a new avenue for treating metastatic liver cancer (Yoo et al., 2017).

#### 3.1.3 Pancreatic Cancer (PC)

Pancreatic cancer has an extremely poor prognosis. Due to its aggressive nature, pancreatic cancer has a 5-year survival rate of less than 10% (Mcguigan et al., 2018), and only 15% of patients are suitable for surgical resection treatment, with the majority of patients progressing to locally advanced stages or developing into metastatic disease (Strobel et al., 2019). Adenovirus and orthopoxvirus effectively suppress tumor metastasis and prolong the survival rate. An animal study has shown that recombinant adenovirus Ad5/3-E2F-d24-vIL2 can counteract immunosuppression and coordinate lymphocyte-mediated immunity (Quixabeira et al., 2021). Adenovirus also aids oncolysis by modulating several signaling pathways, including the CD40, 4-1BB, and ERK signaling pathways. One adenovirus, LOAd703, that carries trimers CD40L and 4-1BBL can initiate and regulate multiple signaling pathways, kill cancer cells, and affect the immune microenvironment (Eriksson et al., 2017). Another recombinant adenovirus, OBP-702, can effectively inhibit the migration of pancreatic ductal adenocarcinoma (PDAC) cells induced by neurosecretory factors by suppressing extracellular regulated protein kinase (ERK) signal transduction in a preclinical, experimental study (Koujima et al., 2020). The application of orthopoxvirus shows that the route of administration impacts oncolytic viruses’ therapeutic effects. An artificially designed immunolytic agent, CF33-hNIS-antiPDL1, had a better curative effect when administered intraperitoneally than intravenous. Early IP treatment has been shown in animal studies to significantly reduce tumor burden, delay disease development, and increase the likelihood of survival (Woo et al., 2020).

VSV is also one of the oncolytic viruses used in pancreatic cancer therapy. However, pancreatic ductal adenocarcinoma cells have a wide range of sensitivity and tolerance to VSV-based oncolytic viruses like VCV-ΔM51 (Felt and Grdzelishvili, 2017). A novel VSV (called VSV-p53OV) derived from the original VSV-p53wt and VSV-p53-CC inhibited PDAC cells from multiplying. However, like other RNA viruses, VSV lacks the proofreading function of the virally encoded RNA-dependent RNA polymerase (RdRp) and is prone to gene mutation [65]. Thus, the genetic stability of the advantageous transgenic fragments carried by VSV is essential. Surprisingly, after 33 generations of virus passages, all viruses retained the original virus-carried p53 (p53wt or p53-CC) and red fluorescent protein (RFP) sequences without any mutations in the transgenic fragment in a basic research study (Seegers et al., 2020).

#### 3.1.4 Gastric Cancer

The herpes simplex virus is the most frequently studied in gastric cancer investigations. However, not all gastric cancer cell lines will respond to the herpes simplex virus transformation. MKN1 is resistant to OHSV expressing platelet lectin-1 (TSP-1) (Tsuji et al., 2013). For MKN1, researchers have developed a new herpes simplex virus called T-SOCS3 that expresses cell signal transduction inhibitor 3 (SOCS-3). In MKN1 cells with low SOCS3 expression after T-01 infection, T-SOCS3 showed a more efficient oncolytic effect in a basic research (Matsumura et al., 2021). In addition, the fourth-generation oncolytic herpes simplex virus (T-hTERT) containing the ICP6 gene, which is regulated by the hTERT promoter, was more toxic to MKN1 cells in an *in-vitro* cellular study (Kato et al., 2021). These studies provide a new strategy for treating drug-resistant gastric cancer cell lines.

Due to the lack of reliable screening strategies and apparent specific clinical manifestations, many patients with gastric cancer present in advanced stages (Smyth et al., 2020). Gastric cancer has a 5-year survival rate of 25-30% (Tirino et al., 2018) and is the third leading cause of cancer-related mortality (Seidlitz et al., 2021). Even in the late stages of gastric cancer, the third-generation herpes simplex virus type 1 (HSV-1) G47Δ can replicate in gastric cancer cell lines, including sclerosing gastric cancer cell lines, and induce cytopathic effects. In addition, G47Δ significantly inhibited tumor growth in an *in-vivo* subcutaneous tumor model, and when administered intratumorally, G47Δ demonstrated good anti-tumor effects regardless of the dose regimen. In a peritoneal dissemination model, intraperitoneal injection of G47Δ also showed significant effectiveness, probably because G47Δ could rapidly penetrate disseminated tumors and selectively replicate therein without the need to inject the virus into each tumor node (Sugawara et al., 2020).

Development of oncolytic adenoviruses to tackle gastric malignancies remain a hot research topic in cancer therapeutics. Rigvir^®^ was the first non-genetically modified gastric cancer virotherapy agent to be approved (Alberts et al., 2016). However, Rigvir^®^’s therapeutic scope is not limited to gastric cancer only. For instance, *in-vitro* experiments–*in-vivo* confirmation is lacking–have shown an inhibitory effect on the viability of multiple tumor cells of human origin, including pancreatic cancer cells (Tilgase et al., 2018). A recent *in-vitro* experiment found that the combination of adenoviruses carrying mK5 and MnSOD genes showed stronger cytotoxicity than a single virus therapy. In addition, no significant differences in body weight were found between the combination treatment mice and the normal mice, demonstrating that this unique combination therapy is plausibly safe (Liu et al., 2022). This strategy of combining two viruses differs from the traditional combination therapies, and it points to a new direction for oncolytic viral research.

#### 3.1.5 Esophageal Cancer

The third-generation oncolytic herpes simplex virus type 1 (HSV-1) g47d might be used to treat a variety of human malignancies, including esophageal cancer (Wang et al., 2014; Wang et al., 2015; Sugawara et al., 2020; Uchihashi et al., 2021). G47d administered intratumorally has been found to have an effective oncolytic effect on subcutaneous and *in-situ* EC tumors in mice. Furthermore, g47d was safe when given orally and intraesophageally in high doses (Yajima et al., 2021). In a tumor-bearing animal model, VSV reduces the development of a variety of cancers when administered intratumorally or intravenously (Felt and Grdzelishvili, 2017), indicating that it might be used to treat esophageal cancer. Although the mechanism by which VSV induces autophagy activity *via* MPS remains unknown, a study found that VSV has oncolytic activity in the esophageal cancer cell line KYSE-30, suggesting that M51R mutant matrix protein (M51R mMP) may promote oncolysis (Douzandegan et al., 2019).

### 3.2 Oncolytic Virus Combined With Chemotherapy

Chemotherapy is one of the most commonly used treatments for cancer. On the other hand, long-term chemotherapy use causes drug resistance, which is accompanied by increasingly severe side effects (Phillips and Mousa, 2022), such as hepatotoxicity and nephrotoxicity, which cause a slew of issues and lower the patient’s quality of life. In recent years, oncolytic viruses paired with chemotherapy have demonstrated many benefits, including reduced toxicity, increased specificity, and improved chemotherapy’s curative efficacy **(** **Table 2****)**.

**Table 2:** Oncolytic viruses combined with chemotherapy.

Type of cancer	Virus	Virus name	Chemotherapeutic drug	Route of virus administration	Effect	Reference
Colorectal cancer	CV	A11	Oxaliplatin	Intratumoral injection	Significantly enhance the efficacy of monotherapy	(Wang et al., 2018)
	Influenza A virus	M1	Lonidamine	i.v.	Infection and tumor killing effect of M1 virus	(Cai et al., 2020)
Liver cancer	NDV	NDV	Fludarabine	i.p.	Synergistic inhibition of tumor growth	(Meng et al., 2019)
		NDV	5-FU	i.p.	Synergistic inhibition of tumor growth	(Assayaghi et al., 2019)
Pancreatic cancer	VV	VV-ING4	Gemcitabine	Intratumoral injection	Synergistic inhibition of tumor growth	(Wu et al., 2017)
	HSV	HF10	Erlotinib, Gemcitabine	Intratumoral injection	Safe treatment for locally advanced pancreatic cancer	(Hirooka et al., 2018)
	ARV	Pelareorep	Gemcitabine	i.v.	Improve the expected survival rate of chemotherapy alone	(Mahalingam et al., 2018)
		Pelareorep	5-FU, Irinotecan, Irinotecan	i.v.	Synergistic inhibition of tumor growth	(Mahalingam et al., 2020)

### 3.3 Colorectal Cancer

One of the most pressing issues in oncolytic viral treatment for colorectal cancer is overcoming chemotherapeutic drug resistance. Although oxaliplatin is one of the first-line chemotherapy drugs for stage III and IV colorectal cancer, patients who are resistant to it have a poor prognosis. Coxsackievirus A11 (CVA11) has a potential oncolytic effect on the sensitivity of the caco-2 cell line to oxaliplatin treatment. However, it has little effect on the oxaliplatin-resistant cell line WiDr. Moreover, the oncolytic activity of CVA11 is enhanced after oxaliplatin pretreatment, but the combination treatment’s cytotoxicity is stronger than either oxaliplatin or CVA11 monotherapy (Wang et al., 2018). It is not by chance that oxaliplatin combined with an oncolytic virus has such a positive impact. Combining lonidamine with the M1 virus shows a synergistic anti-tumor effect. In a mouse colorectal cancer model, lonidamine enhanced the infection and tumor-killing effects of the M1 virus by inhibiting myeloproliferative proto-oncogene (MYC), which not only reveals that lonidamine is a potential synergist of the M1 virus but also implies that MYC deficiency is a potential selective biomarker of M1 virus oncolytic efficacy (Cai et al., 2020). However, it is worth noting that targeted therapeutic combinations do not always improve outcomes. A clinical trial, for example, found that combining lysing euthero virus (Reolysin ™) with standard first-line chemotherapy resulted in a shorter progression-free survival than chemotherapy drugs alone (Jonker et al., 2018).

#### 3.3.1 Liver Cancer

Newcastle disease virus (NDV) mediates innate and adaptive immunity, resulting in a cross-activated anti-tumor immune response (Schwaiger et al., 2017). In addition, its therapeutic effects are associated with CD8 T cells, NK cells, and type I IFNs, but not with CD4 lymphocytes (Zamarin et al., 2014; Ricca et al., 2018). Recent studies have discovered that combining NDV with chemotherapeutic drugs can increase NK cell infiltration and improve anti-tumor effects. Fludarabine, a chemotherapeutic agent for chronic myeloid leukemia, promotes viral replication by targeting signal transducers and activator of transcription 1 (STAT1). This response promotes tumor catabolism and increases ubiquitin proteasomal degradation by accelerating phosphorylated cell signaling with transcriptional activator 3 (p-STAT3) and indoleamine 2,3-dioxygenase-1 (IDO1). Through the mechanisms described above, NDV-mediated viral immunotherapy enhances NK cell infiltration and reduces the number of MDSCs in the tumor immune microenvironment (Meng et al., 2019). NDV has also been demonstrated to improve the anti-tumor action of the chemotherapeutic drug 5-fluorouracil (5-FU) by increasing apoptosis induction. The combination of NDV and 5-FU showed stronger anti-tumor effects than either treatment with NDV or 5-FU alone in an animal study (Assayaghi et al., 2019).

#### 3.3.2 Pancreatic Cancer

Gemcitabine is one of the first-line chemotherapeutic agents for pancreatic cancer. However, due to its increased drug resistance and reduced therapeutic effect, new approaches are needed to improve the efficacy of gemcitabine. Thus, combining gemcitabine with oncolytic viruses may have great anti-tumor therapeutic potential. For example, studies have shown that when gemcitabine (0.01 or 0.05 mM) is combined with oncolytic adenovirus YDC002, the anti-cancer outcomes in pancreatic cancer are better than with a single treatment. This indicates that oncolytic virus YDC002 can make highly resistant pancreatic cancer cells more sensitive to gemcitabine. Besides, it has been found that YDC002 can effectively destroy the extracellular matrix and enhance gemcitabine-induced apoptosis. The safety of gemcitabine and YDC002 combination treatment has been shown to be negligible. Even when compared to gemcitabine or YDC002 alone, this combination therapy treatment did not apparently affect body weight and liver function (Jung et al., 2017).

In addition, gemcitabine can penetrate cancerous cells effectively, and when used in conjunction with other drugs or treatment strategies, beneficial synergistic effects are often reported (Wu et al., 2017). The efficacy and safety of gemcitabine, when combined with other therapies against pancreatic cancer, have also been proven in several clinical studies. For example, a phase I clinical trial found that combining Erlotinib and gemcitabine with direct injection of herpes simplex virus mutant HF10 under ultrasound endoscopic guidance had a stronger anti-tumor effect and that all experimental doses (1×10^6^, 3×10^6^, or 1×10^7^pfu/day×4 times) during dose escalation showed good safety and efficacy (Hirooka et al., 2018). Furthermore, in a phase II clinical trial investigating the role of pelareorep in combination with gemcitabine for advanced pancreatic cancer (the median OS of patients receiving this combination was 10.2 months), the 1- and 2-year survival rates were 45% and 24%, respectively, which were significantly higher than the expected survival rates with gemcitabine therapy alone. This was similar to the efficacy obtained with FOLFIRINOX in the same experimental setting. Pelareorep plus gemcitabine, on the other hand, demonstrated a better safety profile than FOLFIRINOX therapy and could be used as an adjunct to gemcitabine monotherapy (Mahalingam et al., 2018). In a phase Ib clinical study, the combination of pelareorep and pembrolizumab with chemotherapy (5-FU, gemcitabine, or irinotecan) had no significant toxicity despite the desirable efficacy (Mahalingam et al., 2020).

#### 3.3.3 Gastric Cancer

A dual therapy of intraperitoneal injection of green fluorescent protein (GFP)-expressing attenuated adenovirus (OBP-401) plus paclitaxel (PTX) has been demonstrated to successfully suppress peritoneal metastasis of gastric cancer in an *in-situ* xenograft model. Synergistically, OBP-401 and PTX can inhibit the viability of human gastric cancer cells, and PTX enhances OBP-401’s anti-tumor effects by boosting viral replication in cancer cells. However, further research into the combined therapy’s clinical efficacy and tolerance is required (Ishikawa et al., 2020).

### 3.4 Oncolytic Virus Combined With Targeted Therapy

Targeted therapy is one of the most successful ways to treat tumors by inhibiting tumor cell growth or inducing apoptosis by acting on specific cell receptors, signaling, and other channels that promote tumor growth and survival, neovascularization, and cell cycle regulation. However, targeted therapy also has drawbacks, such as drug resistance and side effects, and the use of oncolytic viruses in combination with targeted therapy has improved the therapeutic effect **(** **Table 3****)**.

**Table 3:** Oncolytic viruses combined with targeted therapy.

Type of cancer	Virus	Virus name	Targeted drug	Route of virus administration	Effect	Reference
Colorectal cancer	HSV	CRV	Cetuximab	Intratumoral injection	Synergistic antitumor effect	(Wu et al., 2019)
		T1012G	Propranolol	Intratumoral injection	Synergistic antitumor effect	(Hu et al., 2021a)
		T3855	Trametinib	Intratumoral injection	Synergistic antitumor effect	(Zhou et al., 2021)
	VV	VVL15	PI3K inhibition	i.v.	Synergistic antitumor effect	(Ferguson et al., 2020)
Liver cancer	NDV	NDV	DCA	i.p./i.v.	Synergistic antitumor effect	(Meng et al., 2020)
Pancreatic cancer	VSV	VSV-S	Anti-PD-1 antibody	Intratumoral injection	Synergistic antitumor effect	(Tang et al., 2022)
	AdV	Delta-24-RGD	PS targeted antibody	Intratumoral injection	Synergistic antitumor effect	(Dai et al., 2017)
Gastric cancer	HSV	T1012G	Propranolol	Intratumoral injection	Synergistic antitumor effect	(Hu et al., 2021b)

#### 3.4.1 Colorectal Cancer

EGFR signaling is important for apoptosis, angiogenesis, cell proliferation, migration, and invasion. Cetuximab can enhance the anti-tumor activity of the herpes simplex virus canerpaturev (C-REV) by promoting viral distribution and inhibiting angiogenesis while not interfering with the viral replication process. It is noteworthy that injection of C-REV prior to cetuximab REV has no additive effect on tumor growth compared to C-REV alone in a preclinical study. Therefore, the order of application should be carefully considered (Wu et al., 2019). In addition to cetuximab, the combination therapy of propranolol and oncolytic viruses has been shown to enhance anti-angiogenic effects. Also, propranolol and T1012 combined therapy promoted vascular endothelial growth factor secretion inhibition. However, this combined treatment does not promote viral replication compared to that of cetuximab in an animal study (Hu et al., 2021a).

Furthermore, dysregulation of the mitogen-activated protein kinase (MAPK) signaling cascades occurs frequently in several melanomas. Because of this, the RAS/RAF/MEK/ERK pathway is one of the most studied pathways in cancer biology for the reason that this pathway’s upregulated signaling activity promotes cell proliferation, metastasis, angiogenesis, and others (Degirmenci et al., 2020; Guo et al., 2020). It has been shown that the MEK inhibitor (MEKi) trametinib in combination with OHSV was able to completely eradicate tumors in a CT26 (KRAS-G12D) mouse model. Also, treatment with MEKi promoted OHSV replication in BRAF wt/KRAS mutated tumor cells *in-vitro* (Zhou et al., 2021). In addition to this pathway, IC87114, a selective inhibitor targeting phosphatidylinositol trihydroxyl kinase (PI3K), significantly promoted viral delivery to tumors (Ferguson et al., 2020).

#### 3.4.2 Liver Cancer

The Newcastle disease virus may be useful in the treatment of hepatocellular carcinoma. Unfortunately, due to lactate accumulation, STAT3 activation, IDO1 upregulation, and increased MDSCs infiltration, the Newcastle disease virus causes immunosuppression. The pyruvate dehydrogenase kinase (PDK) inhibitor dichloroacetate (DCA), on the other hand, improves T cell anti-tumor activity by reducing lactate-mediated immunosuppression (Ohashi et al., 2013). This shows that DCA and the Newcastle disease virus can be combined to overcome individual shortfalls. A study report involving DCA and Newcastle disease virus combination treatment against hepatocellular carcinoma indicates that the PDK inhibitor DCA significantly reduced lactate release, STAT3 activation, IDO1 upregulation, and MDSC infiltration in Newcastle disease virus-treated hepatocellular carcinoma. In addition, the presence of DCA boosted Newcastle disease virus replication in hepatocellular carcinoma, implying that DCA improved the anti-tumor immune response to Newcastle disease virus, culminating in the prolonged survival time of tumor-bearing mice (Meng et al., 2020).

#### 3.4.3 Pancreatic Cancer

The programmed death-1 (PD-1) receptor participates in a key pathway of tumor immune escape. Targeted PD-1 immune checkpoint treatment has been approved to treat patients with certain types of malignancies (Tsuruta et al., 2022). In a recent animal study, VSV-S therapy was shown to eliminate tumors in some cases wholly, and it had even better efficacy when combined with anti-PD-1 therapy (Tang et al., 2022).

Phosphatidylserine (PS)-targeted antibodies have shown good efficacy in pancreatic cancer studies and can increase the therapeutic effects of oncolytic viruses against tumors (Digumarti et al., 2014; Chalasani et al., 2015; Freimark et al., 2016). In a mouse PDAC model, elta-24-RGD promotes PS receptor exposure in infected cells, allowing for the application of both elta-24-RGD viral therapy and PS-targeted antibodies in hepatic metastatic pancreatic cancer cells lines. PS-targeting antibodies in combination with elta-24-RGD demonstrated a stronger tumor effect than treatment alone (Dai et al., 2017).

#### 3.4.4 Gastric Cancer

Propranolol coupled with T1012G has a synergistic lethal effect on human and mouse colorectal cancer cells, and also, propranolol has a comparable effect on gastric cancer. Propranolol was discovered to improve the anti-tumor efficacy of viral T1012G in gastric cancer cells by modulating STAT3-PKR-dependent anti-viral responses, which were maintained with type I IFN application, and that β-adrenergic receptor inhibition may provide optimal survival conditions for oncolytic viruses by enhancing intracellular viral replication (Hu et al., 2021b).

### 3.5 Oncolytic Virus Combined With Physical Therapy

#### 3.5.1 Liver Cancer

Radiofrequency ablation (RFA) is the most effective local treatment for early-stage hepatocellular carcinoma. However, it is prone to recurrence when treating medium-to-large tumors. G47Δ, a triple-mutated third-generation oncolytic HSV-1, can enhance RFA efficacy by inducing systemic and specific immunity. The sequential use of G47Δ, RFA, and ICI can further improve the anti-tumor effect (Yamada et al., 2020). Radiofrequency ablation also has a facilitative effect on oncolytic viral therapy. When high doses of T-VEC are injected intratumorally under the guidance of ultrasound and optical imaging techniques, radiofrequency ablation enhances the therapeutic effect of the G47Δ oncolytic virus in a preclinical study. This dual application opens up a new path for treating larger hepatocellular carcinomas locally, while local injection of the virus reduces the viral therapy’s systemic toxicity (Song et al., 2019).

#### 3.5.2 Pancreatic Cancer

Nano blade is a novel tumor ablation technique with the benefits of protecting blood vessels and avoiding heat sink effects. Still, inhomogeneous electric field size is common due to the non-standard distribution of electrode needles or the large size of tumors, therefore, tumors are frequently not wholly resected with nano blade (Kingham et al., 2012). This disadvantage can be avoided by combining it with the use of an oncolytic virus. In a recent study, researchers discovered that combining nano blade and M1 virus inhibited tumor proliferation and significantly prolonged the survival time of *in-situ* immunologically active mouse PC models. On the other hand, the nano blade improves the efficiency of M1 tumor lysis because the extracellular matrix physically limits M1 viruses and immune cells from the vicinity of tumor cells, and the nano blade reduces this impediment by inducing electroporation and T cell immune activation. Thus, the two complement each other and improve tumor treatment (Sun et al., 2021).

### 3.6 Other Combination Therapy With Oncolytic Virus

In addition to the four combination therapy modalities mentioned above, numerous combined treatment strategies have shown promising efficacy in experimental and clinical studies.

In pancreatic cancer, oncolytic viruses paired with chemotherapy have shown promising results, and triple therapy combined with radiotherapy has further boosted therapeutic efficacy. Among multiple combinations of treatment strategies, animals treated with triple combination therapy had the best anti-tumor effect and survival, with one animal experiencing total tumor remission. Although there was no statistically significant difference between the triple combination therapy group and the OAd-hamIFN + radiotherapy group, the significant efficacy of OAd-hamIFN combined with radiotherapy may lead to a reduction in chemotherapy drug dosage. It could be a lifesaver for patients who are unable to tolerate existing chemotherapy treatments (Salzwedel et al., 2018). Through transgenic technology, some success has been made in combating VSV resistance. VSV was combined with ruxolitinib, polybrene, or DEAE-dextran as a new triple therapy to promote VSV attachment and replication and overcome VSV resistance (Felt et al., 2017).

## 4 Conclusions and Challenges

With the development of research on oncolytic virus therapy against malignant tumors of the digestive system, the application of oncolytic viruses in clinical treatment shows remarkable potential. Combined with traditional therapies, oncolytic viruses can have a better anti-tumor effect and show many advantages. First, transgenic oncolytic viruses can assist with the clinical diagnosis of cancers and the detection of disseminated tumor cells. Second, an oncolytic virus and its combination therapy can act on the tumor site more accurately and with stronger specificity. Third, the combination of oncolytic virus therapy and ablation technology improves the limitations of tumor ablation. Fourth, the combination of oncolytic viruses and existing chemotherapeutic drugs can improve the curative effect and reduce the side effects of tumor treatment. Lastly, the application of an oncolytic virus provides a new way to treat unresectable tumors, especially malignant tumors in the special anatomical position of the pancreas.

However, the best route of administration of an oncolytic virus needs to be further studied. At present, there are three main methods: intratumoral injection, intravenous injection, and intraperitoneal injection. Different methods have their own advantages and disadvantages. The intratumoral injection can avoid blood dilution and accurately reach the tumor location, but the limited activation of the systemic immune response weakens tumor cells scattered in other locations. Intravenous injection can be widely used throughout the body and is more convenient, particularly for clinical management (Bommareddy et al., 2018), but its wide range of action does not mean that it can act on target organs, and the effect is greatly reduced (Ferguson et al., 2012). Intraperitoneal injection for tumors in the digestive system seems to achieve more ideal results due to its special anatomical location (Kulu et al., 2009).

In addition, oncolytic viruses and their combination therapies present certain risks. Because of the human anti-viral response, fatigue, fever, chills, leukopenia, and hypotension may occur at the time of oncolytic virotherapy (Andtbacka et al., 2015; Ribas et al., 2016; Gao et al., 2021; Zhang B. et al., 2021), and these adverse effects, although not seriously detrimental to patient health, require the vigilance of researchers and maybe more serious in frail patients. However, oncolytic viral therapy has also caused such serious adverse reactions as grade 3 autoimmune hepatitis, grade 3 aseptic meningitis, and grade 4 pneumonia (Ribas et al., 2017). The treatment strategy should be changed promptly when adverse reactogens are identified. Moreover, the sequential order of combination therapy may affect the treatment efficacy (Wu et al., 2019), and investigators can unfold more research on this aspect. Also, due to the genetic variability of the virus and the imperfect manufacturing and preparation processes, there may be potential risks in the application, and many studies have not yet entered clinical trials. Its clinical efficacy and safety need to be further evaluated.

Therefore, to solve the above problems, we should further explore the drug delivery route of the virus, select the most effective and safe way, optimize the virus preparation process, ensure the stability of the virus, speed up the experimental progress, conduct more extensive and in-depth research, and bring good news to tumor patients as soon as possible.

## Author Contributions

QL, PO, and RG: writing, editing, and visualization. RL and LL: reviewing and editing. XK and QW: conceptualization and supervision. LY: supervision. All authors contributed to the article and approved the submitted version.

## Funding

This study was supported by the Scientific research project of Tianjin Education Commission (2021KJ134), Science and Technology Program of Tianjin (21ZYJDJC00070).

## Conflict of Interest

The authors declare that the research was conducted in the absence of any commercial or financial relationships that could be construed as a potential conflict of interest.

## Publisher’s Note

All claims expressed in this article are solely those of the authors and do not necessarily represent those of their affiliated organizations, or those of the publisher, the editors and the reviewers. Any product that may be evaluated in this article, or claim that may be made by its manufacturer, is not guaranteed or endorsed by the publisher.

## References

[B1] Abdullahi S. Jäkel M. Behrend S. J. Steiger K. Topping G. Krabbe T.. (2018). A Novel Chimeric Oncolytic Virus Vector for Improved Safety and Efficacy as a Platform for the Treatment of Hepatocellular Carcinoma. J. Virol. 92, e01386–18. doi: 10.1128/JVI.01386-18 30232179 PMC6232488

[B2] Aghi M. Rabkin S. D. Martuza R. L. (2007). Angiogenic Response Caused by Oncolytic Herpes Simplex Virus-Induced Reduced Thrombospondin Expression can be Prevented by Specific Viral Mutations or by Administering a Thrombospondin-Derived Peptide. Cancer Res. 67, 440–444. doi: 10.1158/0008-5472.CAN-06-3145 17234749

[B3] Alberts P. Olmane E. Brokāne L. Krastiņa Z. Romanovska M. Kupčs K.. (2016). Long-Term Treatment With the Oncolytic ECHO-7 Virus Rigvir of a Melanoma Stage IV M1c Patient, a Small Cell Lung Cancer Stage IIIA Patient, and a Histiocytic Sarcoma Stage IV Patient-Three Case Reports. Apmis 124, 896–904. doi: 10.1111/apm.12576 27457663

[B4] Al-Ostoot F. H. Salah S. Khamees H. A. Khanum S. A. (2021). Tumor Angiogenesis: Current Challenges and Therapeutic Opportunities. Cancer Treat Res. Commun. 28, 100422. doi: 10.1016/j.ctarc.2021.100422 34147821

[B5] Andtbacka R. H. Kaufman H. L. Collichio F. Amatruda T. Senzer N. Chesney J.. (2015). Talimogene Laherparepvec Improves Durable Response Rate in Patients With Advanced Melanoma. J. Clin. Oncol. 33, 2780–2788. doi: 10.1200/JCO.2014.58.3377 26014293

[B6] Angarita F. A. Acuna S. A. Ottolino-Perry K. Zerhouni S. Mccart J. A. (2013). Mounting a Strategic Offense: Fighting Tumor Vasculature With Oncolytic Viruses. Trends Mol. Med. 19, 378–392. doi: 10.1016/j.molmed.2013.02.008 23540715

[B7] Assayaghi R. M. Alabsi A. M. Swethadri G. Ali A. M. (2019). Liver Pathology in Rats Treated With Newcastle Disease Virus Strains AF2240 and V4-UPM. Asian Pac. J. Cancer Prev. 20, 3071–3075. doi: 10.31557/APJCP.2019.20.10.3071 31653156 PMC6982671

[B8] Axelrod M. L. Cook R. S. Johnson D. B. Balko J. M. (2019). Biological Consequences of MHC-II Expression by Tumor Cells in Cancer. Clin. Cancer Res. 25, 2392–2402. doi: 10.1158/1078-0432.CCR-18-3200 30463850 PMC6467754

[B9] Bernstock J. D. Hoffman S. E. Chen J. A. Gupta S. Kappel A. D. Smith T. R.. (2021). The Current Landscape of Oncolytic Herpes Simplex Viruses as Novel Therapies for Brain Malignancies. Viruses 13, 1158. doi: 10.3390/v13061158 34204248 PMC8234451

[B10] Bommareddy P. K. Shettigar M. Kaufman H. L. (2018). Integrating Oncolytic Viruses in Combination Cancer Immunotherapy. Nat. Rev. Immunol. 18, 498–513. doi: 10.1038/s41577-018-0014-6 29743717

[B11] Borsig L. (2018). Selectins in Cancer Immunity. Glycobiology 28, 648–655. doi: 10.1093/glycob/cwx105 29272415 PMC6711759

[B12] Breitbach C. J. Paterson J. M. Lemay C. G. Falls T. J. Mcguire A. Parato K. A.. (2007). Targeted Inflammation During Oncolytic Virus Therapy Severely Compromises Tumor Blood Flow. Mol. Ther. 15, 1686–1693. doi: 10.1038/sj.mt.6300215 17579581

[B13] Cai J. Zhu W. Lin Y. Hu J. Liu X. Xu W.. (2020). Lonidamine Potentiates the Oncolytic Efficiency of M1 Virus Independent of Hexokinase 2 But *via* Inhibition of Antiviral Immunity. Cancer Cell Int. 20, 532. doi: 10.1186/s12935-020-01598-w 33292203 PMC7607643

[B14] Chalasani P. Marron M. Roe D. Clarke K. Iannone M. Livingston R. B.. (2015). A Phase I Clinical Trial of Bavituximab and Paclitaxel in Patients With HER2 Negative Metastatic Breast Cancer. Cancer Med. 4, 1051–1059. doi: 10.1002/cam4.447 25826750 PMC4529343

[B15] Chen D. Wang R. Long M. Li W. Xiao B. Deng H.. (2021). Identification of *In Vitro* and *In Vivo* Oncolytic Effect in Colorectal Cancer Cells by Orf Virus Strain NA1/11. Oncol. Rep. 45, 535–546. doi: 10.3892/or.2020.7885 33416161 PMC7757097

[B16] Cook M. Chauhan A. (2020). Clinical Application of Oncolytic Viruses: A Systematic Review. Int. J. Mol. Sci. 21, 7505. doi: 10.3390/ijms21207505 PMC7589713 33053757

[B17] Dai B. Roife D. Kang Y. Gumin J. Rios Perez M. V. Li X.. (2017). Preclinical Evaluation of Sequential Combination of Oncolytic Adenovirus Delta-24-RGD and Phosphatidylserine-Targeting Antibody in Pancreatic Ductal Adenocarcinoma. Mol. Cancer Ther. 16, 662–670. doi: 10.1158/1535-7163.MCT-16-0526 28138026 PMC5512885

[B18] Degirmenci U. Wang M. Hu J. (2020). Targeting Aberrant RAS/RAF/MEK/ERK Signaling for Cancer Therapy. Cells 9, 198. doi: 10.3390/cells9010198 PMC7017232 31941155

[B19] Deng H. Liu H. De Silva T. Xue Y. Mohamud Y. Ng C. S.. (2019). Coxsackievirus Type B3 Is a Potent Oncolytic Virus Against KRAS-Mutant Lung Adenocarcinoma. Mol. Ther. Oncol. 14, 266–278. doi: 10.1016/j.omto.2019.07.003 PMC6709373 31463367

[B20] Digumarti R. Bapsy P. P. Suresh A. V. Bhattacharyya G. S. Dasappa L. Shan J. S.. (2014). Bavituximab Plus Paclitaxel and Carboplatin for the Treatment of Advanced non-Small-Cell Lung Cancer. Lung Cancer 86, 231–236. doi: 10.1016/j.lungcan.2014.08.010 25236982

[B21] Douzandegan Y. Tahamtan A. Gray Z. Nikoo H. R. Tabarraei A. Moradi A. (2019). Cell Death Mechanisms in Esophageal Squamous Cell Carcinoma Induced by Vesicular Stomatitis Virus Matrix Protein. Osong Public Health Res. Perspect. 10, 246–252. doi: 10.24171/j.phrp.2019.10.4.08 31497497 PMC6711713

[B22] Eriksson E. Milenova I. Wenthe J. Ståhle M. Leja-Jarblad J. Ullenhag G.. (2017). Shaping the Tumor Stroma and Sparking Immune Activation by CD40 and 4-1BB Signaling Induced by an Armed Oncolytic Virus. Clin. Cancer Res. 23, 5846–5857. doi: 10.1158/1078-0432.CCR-17-0285 28536305

[B23] Felt S. A. Droby G. N. Grdzelishvili V. Z. (2017). Ruxolitinib and Polycation Combination Treatment Overcomes Multiple Mechanisms of Resistance of Pancreatic Cancer Cells to Oncolytic Vesicular Stomatitis Virus. J. Virol. 91, e00461–17. doi: 10.1128/JVI.00461-17 28566376 PMC5533928

[B24] Felt S. A. Grdzelishvili V. Z. (2017). Recent Advances in Vesicular Stomatitis Virus-Based Oncolytic Virotherapy: A 5-Year Update. J. Gen. Virol. 98, 2895–2911. doi: 10.1099/jgv.0.000980 29143726 PMC5845697

[B25] Ferguson M. S. Chard Dunmall L. S. Gangeswaran R. Marelli G. Tysome J. R. Burns E.. (2020). Transient Inhibition of PI3Kδ Enhances the Therapeutic Effect of Intravenous Delivery of Oncolytic Vaccinia Virus. Mol. Ther. 28, 1263–1275. doi: 10.1016/j.ymthe.2020.02.017 32145202 PMC7210704

[B26] Ferguson M. S. Lemoine N. R. Wang Y. (2012). Systemic Delivery of Oncolytic Viruses: Hopes and Hurdles. Adv. Virol. 2012, 805629. doi: 10.1155/2012/805629 22400027 PMC3287020

[B27] Freimark B. D. Gong J. Ye D. Gray M. J. Nguyen V. Yin S.. (2016). Antibody-Mediated Phosphatidylserine Blockade Enhances the Antitumor Responses to CTLA-4 and PD-1 Antibodies in Melanoma. Cancer Immunol. Res. 4, 531–540. doi: 10.1158/2326-6066.CIR-15-0250 27045021 PMC7821912

[B28] Galon J. Bruni D. (2019). Approaches to Treat Immune Hot, Altered and Cold Tumours With Combination Immunotherapies. Nat. Rev. Drug Discovery 18, 197–218. doi: 10.1038/s41573-018-0007-y 30610226

[B29] Gao P. Ding G. Wang L. (2021). The Efficacy and Safety of Oncolytic Viruses in the Treatment of Intermediate to Advanced Solid Tumors: A Systematic Review and Meta-Analysis. Transl. Cancer Res. 10, 4290–4302. doi: 10.21037/tcr-21-905 35116288 PMC8799180

[B30] García-Romero N. Palacín-Aliana I. Esteban-Rubio S. Madurga R. Rius-Rocabert S. Carrión-Navarro J.. (2020). Newcastle Disease Virus (NDV) Oncolytic Activity in Human Glioma Tumors Is Dependent on CDKN2A-Type I IFN Gene Cluster Codeletion. Cells 9, 1405. doi: 10.3390/cells9061405 PMC7349162 32516884

[B31] Gholami S. Marano A. Chen N. G. Aguilar R. J. Frentzen A. Chen C. H.. (2016). Erratum to: A Novel Vaccinia Virus With Dual Oncolytic and Anti-Angiogenic Therapeutic Effects Against Triple-Negative Breast Cancer. Breast Cancer Res. Treat 156, 607–608. doi: 10.1007/s10549-016-3767-2 27026359

[B32] Ghouse S. M. Nguyen H. M. Bommareddy P. K. Guz-Montgomery K. Saha D. (2020). Oncolytic Herpes Simplex Virus Encoding IL12 Controls Triple-Negative Breast Cancer Growth and Metastasis. Front. Oncol. 10, 384. doi: 10.3389/fonc.2020.00384 32266155 PMC7105799

[B33] Gray Z. Tabarraei A. Moradi A. Kalani M. R. (2019). M51R and Delta-M51 Matrix Protein of the Vesicular Stomatitis Virus Induce Apoptosis in Colorectal Cancer Cells. Mol. Biol. Rep. 46, 3371–3379. doi: 10.1007/s11033-019-04799-3 31006094

[B34] Guo Z. S. Lu B. Guo Z. Giehl E. Feist M. Dai E.. (2019). Vaccinia Virus-Mediated Cancer Immunotherapy: Cancer Vaccines and Oncolytics. J. Immunother. Cancer 7, 6. doi: 10.1186/s40425-018-0495-7 30626434 PMC6325819

[B35] Guo Y. J. Pan W. W. Liu S. B. Shen Z. F. Xu Y. Hu L. L. (2020). ERK/MAPK Signalling Pathway and Tumorigenesis. Exp. Ther. Med. 19, 1997–2007. doi: 10.3892/etm.2020.8454 32104259 PMC7027163

[B36] Hamada M. Yura Y. (2020). Efficient Delivery and Replication of Oncolytic Virus for Successful Treatment of Head and Neck Cancer. Int. J. Mol. Sci. 21, 7073. doi: 10.3390/ijms21197073 PMC7582277 32992948

[B37] Hazini A. Dieringer B. Klingel K. Pryshliak M. Geisler A. Kobelt D.. (2021a). Application Route and Immune Status of the Host Determine Safety and Oncolytic Activity of Oncolytic Coxsackievirus B3 Variant PD-H. Viruses 13, 1918. doi: 10.3390/v13101918 34696348 PMC8539752

[B38] Hazini A. Dieringer B. Pryshliak M. Knoch K. P. Heimann L. Tolksdorf B.. (2021b). miR-375- and miR-1-Regulated Coxsackievirus B3 Has No Pancreas and Heart Toxicity But Strong Antitumor Efficiency in Colorectal Carcinomas. Hum. Gene Ther. 32, 216–230. doi: 10.1089/hum.2020.228 33481658

[B39] Hazini A. Pryshliak M. Brückner V. Klingel K. Sauter M. Pinkert S.. (2018). Heparan Sulfate Binding Coxsackievirus B3 Strain PD: A Novel Avirulent Oncolytic Agent Against Human Colorectal Carcinoma. Hum. Gene Ther. 29, 1301–1314. doi: 10.1089/hum.2018.036 29739251

[B40] Hirooka Y. Kasuya H. Ishikawa T. Kawashima H. Ohno E. Villalobos I. B.. (2018). A Phase I Clinical Trial of EUS-Guided Intratumoral Injection of the Oncolytic Virus, HF10 for Unresectable Locally Advanced Pancreatic Cancer. BMC Cancer 18, 596. doi: 10.1186/s12885-018-4453-z 29801474 PMC5970460

[B41] Hu J. Chen C. Lu R. Zhang Y. Wang Y. Hu Q.. (2021a). β-Adrenergic Receptor Inhibitor and Oncolytic Herpesvirus Combination Therapy Shows Enhanced Antitumoral and Antiangiogenic Effects on Colorectal Cancer. Front. Pharmacol. 12, 735278. doi: 10.3389/fphar.2021.735278 34721024 PMC8554205

[B42] Hu J. Lu R. Zhang Y. Li W. Hu Q. Chen C.. (2021b). β-Adrenergic Receptor Inhibition Enhances Oncolytic Herpes Virus Propagation Through STAT3 Activation in Gastric Cancer. Cell Biosci. 11, 174. doi: 10.1186/s13578-021-00687-1 34544479 PMC8454049

[B43] Ishikawa W. Kikuchi S. Ogawa T. Tabuchi M. Tazawa H. Kuroda S.. (2020). Boosting Replication and Penetration of Oncolytic Adenovirus by Paclitaxel Eradicate Peritoneal Metastasis of Gastric Cancer. Mol. Ther. Oncol. 18, 262–271. doi: 10.1016/j.omto.2020.06.021 PMC7378855 32728614

[B44] Jeong S. N. Yoo S. Y. (2020). Novel Oncolytic Virus Armed With Cancer Suicide Gene and Normal Vasculogenic Gene for Improved Anti-Tumor Activity. Cancers (Basel) 12, 1070. doi: 10.3390/cancers12051070 PMC7281019 32344903

[B45] Jia Y. Miyamoto S. Soda Y. Takishima Y. Sagara M. Liao J.. (2019). Extremely Low Organ Toxicity and Strong Antitumor Activity of miR-34-Regulated Oncolytic Coxsackievirus B3. Mol. Ther. Oncol. 12, 246–258. doi: 10.1016/j.omto.2019.01.003 PMC6406029 30891489

[B46] Jośko J. Gwóźdź B. Jedrzejowska-Szypułka H. Hendryk S. (2000). Vascular Endothelial Growth Factor (VEGF) and its Effect on Angiogenesis. Med. Sci. Monit. 6, 1047–1052. doi: 10.1007/978-3-319-28140-7_58 11208453

[B47] Johnstone R. W. Frew A. J. Smyth M. J. (2008). The TRAIL Apoptotic Pathway in Cancer Onset, Progression and Therapy. Nat. Rev. Cancer 8, 782–798. doi: 10.1038/nrc2465 18813321

[B48] Jonker D. J. Tang P. A. Kennecke H. Welch S. A. Cripps M. C. Asmis T.. (2018). A Randomized Phase II Study of FOLFOX6/Bevacizumab With or Without Pelareorep in Patients With Metastatic Colorectal Cancer: IND.210, a Canadian Cancer Trials Group Trial. Clin. Colorectal Cancer 17, 231–239.e237. doi: 10.1016/j.clcc.2018.03.001 29653857

[B49] Jung K. H. Choi I. K. Lee H. S. Yan H. H. Son M. K. Ahn H. M.. (2017). Oncolytic Adenovirus Expressing Relaxin (YDC002) Enhances Therapeutic Efficacy of Gemcitabine Against Pancreatic Cancer. Cancer Lett. 396, 155–166. doi: 10.1016/j.canlet.2017.03.009 28315430

[B50] Katayama Y. Tachibana M. Kurisu N. Oya Y. Terasawa Y. Goda H.. (2018). Oncolytic Reovirus Inhibits Immunosuppressive Activity of Myeloid-Derived Suppressor Cells in a TLR3-Dependent Manner. J. Immunol. 200, 2987–2999. doi: 10.4049/jimmunol.1700435 29555782

[B51] Kato T. Nakamori M. Matsumura S. Nakamura M. Ojima T. Fukuhara H.. (2021). Oncolytic Virotherapy With Human Telomerase Reverse Transcriptase Promoter Regulation Enhances Cytotoxic Effects Against Gastric Cancer. Oncol. Lett. 21, 490. doi: 10.3892/ol.2021.12751 33968206 PMC8100961

[B52] Kingham T. P. Karkar A. M. D'angelica M. I. Allen P. J. Dematteo R. P. Getrajdman G. I.. (2012). Ablation of Perivascular Hepatic Malignant Tumors With Irreversible Electroporation. J. Am. Coll. Surg. 215, 379–387. doi: 10.1016/j.jamcollsurg.2012.04.029 22704820

[B53] Koujima T. Tazawa H. Ieda T. Araki H. Fushimi T. Shoji R.. (2020). Oncolytic Virus-Mediated Targeting of the ERK Signaling Pathway Inhibits Invasive Propensity in Human Pancreatic Cancer. Mol. Ther. Oncol. 17, 107–117. doi: 10.1016/j.omto.2020.03.016 PMC7163052 32322667

[B54] Kozak R. A. Hattin L. Biondi M. J. Corredor J. C. Walsh S. Xue-Zhong M.. (2017). Replication and Oncolytic Activity of an Avian Orthoreovirus in Human Hepatocellular Carcinoma Cells. Viruses 9, 90. doi: 10.3390/v9040090 PMC5408696 28441762

[B55] Kulu Y. Dorfman J. D. Kuruppu D. Fuchs B. C. Goodwin J. M. Fujii T.. (2009). Comparison of Intravenous Versus Intraperitoneal Administration of Oncolytic Herpes Simplex Virus 1 for Peritoneal Carcinomatosis in Mice. Cancer Gene Ther. 16, 291–297. doi: 10.1038/cgt.2008.83 18989355 PMC2657185

[B56] Kurozumi K. Hardcastle J. Thakur R. Shroll J. Nowicki M. Otsuki A.. (2008). Oncolytic HSV-1 Infection of Tumors Induces Angiogenesis and Upregulates CYR61. Mol. Ther. 16, 1382–1391. doi: 10.1038/mt.2008.112 18545226 PMC2659780

[B57] Lawler S. E. Speranza M. C. Cho C. F. Chiocca E. A. (2017). Oncolytic Viruses in Cancer Treatment: A Review. JAMA Oncol. 3, 841–849. doi: 10.1001/jamaoncol.2016.2064 27441411

[B58] Lin Y. Zhang H. Liang J. Li K. Zhu W. Fu L.. (2014). Identification and Characterization of Alphavirus M1 as a Selective Oncolytic Virus Targeting ZAP-Defective Human Cancers. Proc. Natl. Acad. Sci. U.S.A. 111, E4504–E4512. doi: 10.1073/pnas.1408759111 25288727 PMC4210284

[B59] Liu Y. Cai J. Liu W. Lin Y. Guo L. Liu X.. (2020). Intravenous Injection of the Oncolytic Virus M1 Awakens Antitumor T Cells and Overcomes Resistance to Checkpoint Blockade. Cell Death Dis. 11, 1062. doi: 10.1038/s41419-020-03285-0 33311488 PMC7733593

[B60] Liu S. S. Hu J. Q. Gu J. F. Ni A. M. Tang W. H. Liu X. Y. (2022). Combined Oncolytic Adenovirus Carrying MnSOD and Mk5 Genes Both Regulated by Survivin Promoter has a Synergistic Inhibitory Effect on Gastric Cancer. Neoplasma 69, 36–48. doi: 10.4149/neo_2021_210508N624 34881625

[B61] Lugano R. Ramachandran M. Dimberg A. (2020). Tumor Angiogenesis: Causes, Consequences, Challenges and Opportunities. Cell Mol. Life Sci. 77, 1745–1770. doi: 10.1007/s00018-019-03351-7 31690961 PMC7190605

[B62] Mahalingam D. Goel S. Aparo S. Patel Arora S. Noronha N. Tran H.. (2018). A Phase II Study of Pelareorep (REOLYSIN(®)) in Combination With Gemcitabine for Patients With Advanced Pancreatic Adenocarcinoma. Cancers (Basel) 10, 160. doi: 10.3390/cancers10060160 PMC6025223 29799479

[B63] Mahalingam D. Wilkinson G. A. Eng K. H. Fields P. Raber P. Moseley J. L.. (2020). Pembrolizumab in Combination With the Oncolytic Virus Pelareorep and Chemotherapy in Patients With Advanced Pancreatic Adenocarcinoma: A Phase Ib Study. Clin. Cancer Res. 26, 71–81. doi: 10.1158/1078-0432.CCR-19-2078 31694832 PMC6942612

[B64] Ma J. Ramachandran M. Jin C. Quijano-Rubio C. Martikainen M. Yu D.. (2020). Characterization of Virus-Mediated Immunogenic Cancer Cell Death and the Consequences for Oncolytic Virus-Based Immunotherapy of Cancer. Cell Death Dis. 11, 48. doi: 10.1038/s41419-020-2236-3 31969562 PMC6976683

[B65] Matsumura S. Nakamori M. Tsuji T. Kato T. Nakamura M. Ojima T.. (2021). Oncolytic Virotherapy With SOCS3 Enhances Viral Replicative Potency and Oncolysis for Gastric Cancer. Oncotarget 12, 344–354. doi: 10.18632/oncotarget.27873 33659045 PMC7899552

[B66] Mcguigan A. Kelly P. Turkington R. C. Jones C. Coleman H. G. Mccain R. S. (2018). Pancreatic Cancer: A Review of Clinical Diagnosis, Epidemiology, Treatment and Outcomes. World J. Gastroenterol. 24, 4846–4861. doi: 10.3748/wjg.v24.i43.4846 30487695 PMC6250924

[B67] Meng G. Fei Z. Fang M. Li B. Chen A. Xu C.. (2019). Fludarabine as an Adjuvant Improves Newcastle Disease Virus-Mediated Antitumor Immunity in Hepatocellular Carcinoma. Mol. Ther. Oncol. 13, 22–34. doi: 10.1016/j.omto.2019.03.004 PMC6461577 31011625

[B68] Meng G. Li B. Chen A. Zheng M. Xu T. Zhang H.. (2020). Targeting Aerobic Glycolysis by Dichloroacetate Improves Newcastle Disease Virus-Mediated Viro-Immunotherapy in Hepatocellular Carcinoma. Br. J. Cancer 122, 111–120. doi: 10.1038/s41416-019-0639-7 31819179 PMC6964686

[B69] Mondal M. Guo J. He P. Zhou D. (2020). Recent Advances of Oncolytic Virus in Cancer Therapy. Hum. Vaccin Immunother. 16, 2389–2402. doi: 10.1080/21645515.2020.1723363 32078405 PMC7644205

[B70] Nair M. Bolyard C. Lee T. J. Kaur B. Yoo J. Y. (2021). Therapeutic Application of Brain-Specific Angiogenesis Inhibitor 1 for Cancer Therapy. Cancers (Basel) 13, 3562. doi: 10.3390/cancers13143562 34298774 PMC8303278

[B71] Nakao S. Arai Y. Tasaki M. Yamashita M. Murakami R. Kawase T.. (2020). Intratumoral Expression of IL-7 and IL-12 Using an Oncolytic Virus Increases Systemic Sensitivity to Immune Checkpoint Blockade. Sci. Transl. Med. 12, eaax7992. doi: 10.1126/scitranslmed.aax7992 31941828

[B72] Ohashi T. Akazawa T. Aoki M. Kuze B. Mizuta K. Ito Y.. (2013). Dichloroacetate Improves Immune Dysfunction Caused by Tumor-Secreted Lactic Acid and Increases Antitumor Immunoreactivity. Int. J. Cancer 133, 1107–1118. doi: 10.1002/ijc.28114 23420584

[B73] Parker B. S. Rautela J. Hertzog P. J. (2016). Antitumour Actions of Interferons: Implications for Cancer Therapy. Nat. Rev. Cancer 16, 131–144. doi: 10.1038/nrc.2016.14 26911188

[B74] Passaro C. Borriello F. Vastolo V. Di Somma S. Scamardella E. Gigantino V.. (2016). The Oncolytic Virus Dl922-947 Reduces IL-8/CXCL8 and MCP-1/CCL2 Expression and Impairs Angiogenesis and Macrophage Infiltration in Anaplastic Thyroid Carcinoma. Oncotarget 7, 1500–1515. doi: 10.18632/oncotarget.6430 26625205 PMC4811476

[B75] Phillips M. C. Mousa S. A. (2022) Clinical Application of Nano-Targeting for Enhancing Chemotherapeutic Efficacy and Safety in Cancer Management Nanomed. (Lond). 17, 405–21 doi: 10.2217/nnm-2021-0361 35118878

[B76] Pryshliak M. Hazini A. Knoch K. Dieringer B. Tolksdorf B. Solimena M.. (2020). MiR-375-Mediated Suppression of Engineered Coxsackievirus B3 in Pancreatic Cells. FEBS Lett. 594, 763–775. doi: 10.1002/1873-3468.13647 31643074

[B77] Qian C. N. Pezzella F. (2018). Tumor Vasculature: A Sally Port for Inhibiting Cancer Cell Spreading. Cancer Commun. (Lond). 38, 52. doi: 10.1186/s40880-018-0322-z 30075743 PMC6076415

[B78] Quixabeira D. C. A. Zafar S. Santos J. M. Cervera-Carrascon V. Havunen R. Kudling T. V.. (2021). Oncolytic Adenovirus Coding for a Variant Interleukin 2 (vIL-2) Cytokine Re-Programs the Tumor Microenvironment and Confers Enhanced Tumor Control. Front. Immunol. 12, 674400. doi: 10.3389/fimmu.2021.674400 34084172 PMC8168464

[B79] Raja J. Ludwig J. M. Gettinger S. N. Schalper K. A. Kim H. S. (2018). Oncolytic Virus Immunotherapy: Future Prospects for Oncology. J. Immunother. Cancer 6, 140. doi: 10.1186/s40425-018-0458-z 30514385 PMC6280382

[B80] Ramamurthy N. Pathak D. C. D'silva A. L. Batheja R. Mariappan A. K. Vakharia V. N.. (2021). Evaluation of the Oncolytic Property of Recombinant Newcastle Disease Virus Strain R2B in 4T1 and B16-F10 Cells *in-Vitro* . Res. Vet. Sci. 139, 159–165. doi: 10.1016/j.rvsc.2021.07.028 34332418

[B81] Ramelyte E. Tastanova A. Balázs Z. Ignatova D. Turko P. Menzel U.. (2021). Oncolytic Virotherapy-Mediated Anti-Tumor Response: A Single-Cell Perspective. Cancer Cell 39, 394–406.e394. doi: 10.1016/j.ccell.2020.12.022 33482123

[B82] Ribas A. Dummer R. Puzanov I. Vanderwalde A. Andtbacka R. H. I. Michielin O.. (2017). Oncolytic Virotherapy Promotes Intratumoral T Cell Infiltration and Improves Anti-PD-1 Immunotherapy. Cell 170, 1109–1119.e1110. doi: 10.1016/j.cell.2017.08.027 28886381 PMC8034392

[B83] Ribas A. Hamid O. Daud A. Hodi F. S. Wolchok J. D. Kefford R.. (2016). Association of Pembrolizumab With Tumor Response and Survival Among Patients With Advanced Melanoma. Jama 315, 1600–1609. doi: 10.1001/jama.2016.4059 27092830

[B84] Ricca J. M. Oseledchyk A. Walther T. Liu C. Mangarin L. Merghoub T.. (2018). Pre-Existing Immunity to Oncolytic Virus Potentiates Its Immunotherapeutic Efficacy. Mol. Ther. 26, 1008–1019. doi: 10.1016/j.ymthe.2018.01.019 29478729 PMC6079372

[B85] Sahin T. T. Kasuya H. Nomura N. Shikano T. Yamamura K. Gewen T.. (2012). Impact of Novel Oncolytic Virus HF10 on Cellular Components of the Tumor Microenviroment in Patients With Recurrent Breast Cancer. Cancer Gene Ther. 19, 229–237. doi: 10.1038/cgt.2011.80 22193629

[B86] Salzwedel A. O. Han J. Larocca C. J. Shanley R. Yamamoto M. Davydova J. (2018). Combination of Interferon-Expressing Oncolytic Adenovirus With Chemotherapy and Radiation is Highly Synergistic in Hamster Model of Pancreatic Cancer. Oncotarget 9, 18041–18052. doi: 10.18632/oncotarget.24710 29719589 PMC5915056

[B87] Schwaiger T. Knittler M. R. Grund C. Roemer-Oberdoerfer A. Kapp J. F. Lerch M. M.. (2017). Newcastle Disease Virus Mediates Pancreatic Tumor Rejection *via* NK Cell Activation and Prevents Cancer Relapse by Prompting Adaptive Immunity. Int. J. Cancer 141, 2505–2516. doi: 10.1002/ijc.31026 28857157

[B88] Seegers S. L. Frasier C. Greene S. Nesmelova I. V. Grdzelishvili V. Z. (2020). Experimental Evolution Generates Novel Oncolytic Vesicular Stomatitis Viruses With Improved Replication in Virus-Resistant Pancreatic Cancer Cells. J. Virol. 94, e01643–19. doi: 10.1128/JVI.01643-19 31694943 PMC7000975

[B89] Seidlitz T. Koo B. K. Stange D. E. (2021). Gastric Organoids-an *In Vitro* Model System for the Study of Gastric Development and Road to Personalized Medicine. Cell Death Differ. 28, 68–83. doi: 10.1038/s41418-020-00662-2 33223522 PMC7852679

[B90] Simmonds P. Gorbalenya A. E. Harvala H. Hovi T. Knowles N. J. Lindberg A. M.. (2020). Recommendations for the Nomenclature of Enteroviruses and Rhinoviruses. Arch. Virol. 165, 793–797. doi: 10.1007/s00705-019-04520-6 31980941 PMC7024059

[B91] Smyth E. C. Nilsson M. Grabsch H. I. Van Grieken N. C. Lordick F. (2020). Gastric Cancer. Lancet 396, 635–648. doi: 10.1016/S0140-6736(20)31288-5 32861308

[B92] Song J. Zhang F. Ji J. Chen M. Li Q. Weng Q.. (2019). Orthotopic Hepatocellular Carcinoma: Molecular Imaging-Monitored Intratumoral Hyperthermia-Enhanced Direct Oncolytic Virotherapy. Int. J. Hyperthermia 36, 344–350. doi: 10.1080/02656736.2019.1569731 30776922 PMC6988576

[B93] Steinhauer D. A. Domingo E. Holland J. J. (1992). Lack of evidence for proofreading mechanisms associated with an RNA virus polymerase. Gene 122, 281–288. 1336756 10.1016/0378-1119(92)90216-c

[B94] Strobel O. Neoptolemos J. Jäger D. Büchler M. W. (2019). Optimizing the Outcomes of Pancreatic Cancer Surgery. Nat. Rev. Clin. Oncol. 16, 11–26. doi: 10.1038/s41571-018-0112-1 30341417

[B95] Sugawara K. Iwai M. Yajima S. Tanaka M. Yanagihara K. Seto Y.. (2020). Efficacy of a Third-Generation Oncolytic Herpes Virus G47Δ in Advanced Stage Models of Human Gastric Cancer. Mol. Ther. Oncol. 17, 205–215. doi: 10.1016/j.omto.2020.03.022 PMC7178322 32346610

[B96] Sung H. Ferlay J. Siegel R. L. Laversanne M. Soerjomataram I. Jemal A.. (2021). Global Cancer Statistics 2020: GLOBOCAN Estimates of Incidence and Mortality Worldwide for 36 Cancers in 185 Countries. CA Cancer J. Clin. 71, 209–249. doi: 10.3322/caac.21660 33538338

[B97] Sun S. Liu Y. He C. Hu W. Liu W. Huang X.. (2021). Combining NanoKnife With M1 Oncolytic Virus Enhances Anticancer Activity in Pancreatic Cancer. Cancer Lett. 502, 9–24. doi: 10.1016/j.canlet.2020.12.018 33444691

[B98] Tang S. Shi L. Luker B. T. Mickler C. Suresh B. Lesinski G. B.. (2022). Modulation of the Tumor Microenvironment by Armed Vesicular Stomatitis Virus in a Syngeneic Pancreatic Cancer Model. Virol. J. 19, 32. doi: 10.1186/s12985-022-01757-7 35197076 PMC8867845

[B99] Tian Y. Yao W. He D. Xu Y. Li Y. Zhu Y.. (2020). A Dual Cancer-Specific Recombinant Adenovirus Suppresses the Growth of Liver Cancer Cells *In Vivo* and *In Vitro* . Anticancer Drugs 31, 110–122. doi: 10.1097/CAD.0000000000000854 31658131

[B100] Tilgase A. Patetko L. Blāķe I. Ramata-Stunda A. Borodušķis M. Alberts P. (2018). Effect of the Oncolytic ECHO-7 Virus Rigvir® on the Viability of Cell Lines of Human Origin *In Vitro* . J. Cancer 9, 1033–1049. doi: 10.7150/jca.23242 29581783 PMC5868171

[B101] Tirino G. Pompella L. Petrillo A. Laterza M. M. Pappalardo A. Caterino M.. (2018). What's New in Gastric Cancer: The Therapeutic Implications of Molecular Classifications and Future Perspectives. Int. J. Mol. Sci. 19, 2659. doi: 10.3390/ijms19092659 PMC6165492 30205505

[B102] Tsuji T. Nakamori M. Iwahashi M. Nakamura M. Ojima T. Iida T.. (2013). An Armed Oncolytic Herpes Simplex Virus Expressing Thrombospondin-1 has an Enhanced *In Vivo* Antitumor Effect Against Human Gastric Cancer. Int. J. Cancer 132, 485–494. doi: 10.1002/ijc.27681 22729516

[B103] Tsuruta A. Shiiba Y. Matsunaga N. Fujimoto M. Yoshida Y. Koyanagi S.. (2022). Diurnal Expression of PD-1 on Tumor-Associated Macrophages Underlies the Dosing Time-Dependent Anti-Tumor Effects of the PD-1/PD-L1 Inhibitor BMS-1 in B16/BL6 Melanoma-Bearing Mice. Mol. Cancer Res molcanres MCR-21-0786-E.2021. doi: 10.1158/1541-7786.MCR-21-0786 PMC9381128 35190830

[B104] Uchihashi T. Nakahara H. Fukuhara H. Iwai M. Ito H. Sugauchi A.. (2021). Oncolytic Herpes Virus G47Δ Injected Into Tongue Cancer Swiftly Traffics in Lymphatics and Suppresses Metastasis. Mol. Ther. Oncol. 22, 388–398. doi: 10.1016/j.omto.2021.06.008 PMC8430046 34553027

[B105] Wang J. Y. Chen H. Dai S. Z. Huang F. Y. Lin Y. Y. Wang C. C.. (2022). Immunotherapy Combining Tumor and Endothelium Cell Lysis With Immune Enforcement by Recombinant MIP-3α Newcastle Disease Virus in a Vessel-Targeting Liposome Enhances Antitumor Immunity. J. Immunother. Cancer 10, e003950. doi: 10.1136/jitc-2021-003950 35256516 PMC8905871

[B106] Wang G. Kang X. Chen K. S. Jehng T. Jones L. Chen J.. (2020). An Engineered Oncolytic Virus Expressing PD-L1 Inhibitors Activates Tumor Neoantigen-Specific T Cell Responses. Nat. Commun. 11, 1–14. doi: 10.1038/s41467-020-15229-5 32170083 PMC7070065

[B107] Wang B. Ogata H. Takishima Y. Miyamoto S. Inoue H. Kuroda M.. (2018). ). A Novel Combination Therapy for Human Oxaliplatin-Resistant Colorectal Cancer Using Oxaliplatin and Coxsackievirus A11. Anticancer Res. 38, 6121–6126. doi: 10.21873/anticanres.12963 30396927

[B108] Wang N. Wang J. Zhang Z. Cao H. Yan W. Chu Y.. (2021). A Novel Vaccinia Virus Enhances Anti-Tumor Efficacy and Promotes a Long-Term Anti-Tumor Response in a Murine Model of Colorectal Cancer. Mol. Ther. Oncol. 20, 71–81. doi: 10.1016/j.omto.2020.11.002 PMC7851495 33575472

[B109] Wang J. N. Xu L. H. Zeng W. G. Hu P. Rabkin S. D. Liu R. R. (2015). Treatment of Human Thyroid Carcinoma Cells With the G47delta Oncolytic Herpes Simplex Virus. Asian Pac. J. Cancer Prev. 16, 1241–1245. doi: 10.7314/APJCP.2015.16.3.1241 25735362

[B110] Wang J. Xu L. Zeng W. Hu P. Zeng M. Rabkin S. D.. (2014). Treatment of Human Hepatocellular Carcinoma by the Oncolytic Herpes Simplex Virus G47delta. Cancer Cell Int. 14, 83. doi: 10.1186/s12935-014-0083-y 25360068 PMC4213511

[B111] Wang Z. Yu B. Wang B. Yan J. Feng X. Wang Z.. (2016). A Novel Capsid-Modified Oncolytic Recombinant Adenovirus Type 5 for Tumor-Targeting Gene Therapy by Intravenous Route. Oncotarget 7, 47287–47301. doi: 10.18632/oncotarget.10075 27323824 PMC5216942

[B112] Warner S. G. Kim S. I. Chaurasiya S. O'leary M. P. Lu J. Sivanandam V.. (2019). A Novel Chimeric Poxvirus Encoding hNIS Is Tumor-Tropic, Imageable, and Synergistic With Radioiodine to Sustain Colon Cancer Regression. Mol. Ther. Oncol. 13, 82–92. doi: 10.1016/j.omto.2019.04.001 PMC6495072 31061881

[B113] Woo Y. Zhang Z. Yang A. Chaurasiya S. Park A. K. Lu J.. (2020). Novel Chimeric Immuno-Oncolytic Virus CF33-hNIS-Antipdl1 for the Treatment of Pancreatic Cancer. J. Am. Coll. Surg. 230, 709–717. doi: 10.1016/j.jamcollsurg.2019.12.027 32032721 PMC8787938

[B114] Wu Z. Ichinose T. Naoe Y. Matsumura S. Villalobos I. B. Eissa I. R.. (2019). Combination of Cetuximab and Oncolytic Virus Canerpaturev Synergistically Inhibits Human Colorectal Cancer Growth. Mol. Ther. Oncol. 13, 107–115. doi: 10.1016/j.omto.2019.04.004 PMC6539424 31193737

[B115] Wu Y. Mou X. Wang S. Liu X. E. Sun X. (2017). ING4 Expressing Oncolytic Vaccinia Virus Promotes Anti-Tumor Efficiency and Synergizes With Gemcitabine in Pancreatic Cancer. Oncotarget 8, 82728–82739. doi: 10.18632/oncotarget.21095 29137298 PMC5669924

[B116] Yajima S. Sugawara K. Iwai M. Tanaka M. Seto Y. Todo T. (2021). Efficacy and Safety of a Third-Generation Oncolytic Herpes Virus G47Δ in Models of Human Esophageal Carcinoma. Mol. Ther. Oncol. 23, 402–411. doi: 10.1016/j.omto.2021.10.012 PMC8605086 34853811

[B117] Yamada T. Tateishi R. Iwai M. Koike K. Todo T. (2020). Neoadjuvant Use of Oncolytic Herpes Virus G47Δ Enhances the Antitumor Efficacy of Radiofrequency Ablation. Mol. Ther. Oncol. 18, 535–545. doi: 10.1016/j.omto.2020.08.010 PMC7501409 32995479

[B118] Yang L. Gu X. Yu J. Ge S. Fan X. (2021). Oncolytic Virotherapy: From Bench to Bedside. Front. Cell Dev. Biol. 9, 790150. doi: 10.3389/fcell.2021.790150 34901031 PMC8662562

[B119] Ye T. Jiang K. Wei L. Barr M. P. Xu Q. Zhang G.. (2018). Oncolytic Newcastle Disease Virus Induces Autophagy-Dependent Immunogenic Cell Death in Lung Cancer Cells. Am. J. Cancer Res. 8, 1514–1527. 30210920 PMC6129498

[B120] Ye Z. Wang X. Hao S. Zhong J. Xiang J. Yang J. (2006). Oncolytic Adenovirus-Mediated E1A Gene Therapy Induces Tumor-Cell Apoptosis and Reduces Tumor Angiogenesis Leading to Inhibition of Hepatocellular Carcinoma Growth in Animal Model. Cancer Biother. Radiopharm. 21, 225–234. doi: 10.1089/cbr.2006.21.225 16918299

[B121] Ying L. Cheng H. Xiong X. W. Yuan L. Peng Z. H. Wen Z. W.. (2017). Interferon Alpha Antagonizes the Anti-Hepatoma Activity of the Oncolytic Virus M1 by Stimulating Anti-Viral Immunity. Oncotarget 8, 24694–24705. doi: 10.18632/oncotarget.15788 28445966 PMC5421880

[B122] Yoo S. Y. Jeong S. N. Kang D. H. Heo J. (2017). Evolutionary Cancer-Favoring Engineered Vaccinia Virus for Metastatic Hepatocellular Carcinoma. Oncotarget 8, 71489–71499. doi: 10.18632/oncotarget.17288 29069721 PMC5641064

[B123] Zamarin D. Holmgaard R. B. Subudhi S. K. Park J. S. Mansour M. Palese P.. (2014). Localized Oncolytic Virotherapy Overcomes Systemic Tumor Resistance to Immune Checkpoint Blockade Immunotherapy. Sci. Transl. Med. 6, 226ra232. doi: 10.1126/scitranslmed.3008095 PMC4106918 24598590

[B124] Zautner A. E. Körner U. Henke A. Badorff C. Schmidtke M. (2003). Heparan Sulfates and Coxsackievirus-Adenovirus Receptor: Each One Mediates Coxsackievirus B3 PD Infection. J. Virol. 77, 10071–10077. doi: 10.1128/JVI.77.18.10071-10077.2003 12941917 PMC224569

[B125] Zhang B. Huang J. Tang J. Hu S. Luo S. Luo Z.. (2021). Intratumoral OH2, an Oncolytic Herpes Simplex Virus 2, in Patients With Advanced Solid Tumors: A Multicenter, Phase I/II Clinical Trial. J. Immunother. Cancer 9, e002224 doi: 10.1136/jitc-2020-002224 33837053 PMC8043042

[B126] Zhang W. Hu X. Liang J. Zhu Y. Zeng B. Feng L.. (2020). Ohsv2 Can Target Murine Colon Carcinoma by Altering the Immune Status of the Tumor Microenvironment and Inducing Antitumor Immunity. Mol. Ther. Oncol. 16, 158–171. doi: 10.1016/j.omto.2019.12.012 PMC7011019 32055679

[B127] Zhang W. Zeng B. Hu X. Zou L. Liang J. Song Y.. (2021). Oncolytic Herpes Simplex Virus Type 2 Can Effectively Inhibit Colorectal Cancer Liver Metastasis by Modulating the Immune Status in the Tumor Microenvironment and Inducing Specific Antitumor Immunity. Hum. Gene Ther. 32, 203–215. doi: 10.1089/hum.2020.239 33176492

[B128] Zhou X. Zhao J. Zhang J. V. Wu Y. Wang L. Chen X.. (2021). Enhancing Therapeutic Efficacy of Oncolytic Herpes Simplex Virus With MEK Inhibitor Trametinib in Some BRAF or KRAS-Mutated Colorectal or Lung Carcinoma Models. Viruses 13, 1758. doi: 10.3390/v13091758 34578339 PMC8473197

[B129] Zitvogel L. Galluzzi L. Kepp O. Smyth M. J. Kroemer G. (2015). Type I Interferons in Anticancer Immunity. Nat. Rev. Immunol. 15, 405–414. doi: 10.1038/nri3845 26027717

